# Parasubthalamic calretinin neurons modulate wakefulness associated with exploration in male mice

**DOI:** 10.1038/s41467-023-37797-y

**Published:** 2023-04-24

**Authors:** Han Guo, Jian-Bo Jiang, Wei Xu, Mu-Tian Zhang, Hui Chen, Huan-Ying Shi, Lu Wang, Miao He, Michael Lazarus, Shan-Qun Li, Zhi-Li Huang, Wei-Min Qu

**Affiliations:** 1 Department of Pharmacology, School of Basic Medical Sciences; State Key Laboratory of Medical Neurobiology and MOE Frontiers Center for Brain Science, and Institutes of Brain Science, Fudan University, Shanghai, 200032 China; 2 Department of Pulmonary Medicine, Zhongshan Hospital, Fudan University, Shanghai, 200032 China; 3 Department of Pharmacy, Huadong Hospital, Fudan University, Shanghai, China; 4 Department of Pharmacy, Huashan Hospital, Fudan University, Shanghai, China; 5 International Institute for Integrative Sleep Medicine (WPIIIIS), University of Tsukuba, Tsukuba, Ibaraki 305-8575 Japan

**Keywords:** Non-REM sleep, Neural circuits, Wakefulness

## Abstract

The parasubthalamic nucleus (PSTN) is considered to be involved in motivation, feeding and hunting, all of which are highly depending on wakefulness. However, the roles and underlying neural circuits of the PSTN in wakefulness remain unclear. Neurons expressing calretinin (CR) account for the majority of PSTN neurons. In this study in male mice, fiber photometry recordings showed that the activity of PSTN^CR^ neurons increased at the transitions from non-rapid eye movement (non-REM, NREM) sleep to either wakefulness or REM sleep, as well as exploratory behavior. Chemogenetic and optogenetic experiments demonstrated that PSTN^CR^ neurons were necessary for initiating and/or maintaining arousal associated with exploration. Photoactivation of projections of PSTN^CR^ neurons revealed that they regulated exploration-related wakefulness by innervating the ventral tegmental area. Collectively, our findings indicate that PSTN^CR^ circuitry is essential for the induction and maintenance of the awake state associated with exploration.

## Abstract (web summary)

The neural circuits regulating wakefulness have not been fully resolved. Here, the authors reveal that neurons expressing calretinin in the parasubthalamic nucleus play a key role in the induction and maintenance of the awake state associated with exploration via projections to the ventral tegmental area.

## Introduction

The maintenance of curiosity and the exploration of surroundings are vital to survival for mice. Exploratory behaviors, including sniffing and rearing, are prerequisites for mice to acquire and analyze information about the surrounding environment, and these behaviors are highly dependent on wakefulness. However, the neural circuits and action mechanisms through which the brain regulates wakefulness associated with exploration have not been fully resolved.

Ventral tegmental area (VTA) dopamine neurons play a fundamental role in wakefulness and exploration-based social interaction^1,2^. Monosynaptic retrograde tracing experiments show that the parasubthalamic nucleus (PSTN) is the densest input in the hypothalamus to the VTA dopaminergic neurons^3^. The PSTN also has dense connections with the parabrachial nucleus (PB)^4^ and the paraventricular thalamus (PVT)^5,6^, and both the PB and PVT have a strong ability to control wakefulness^7–12^. The PSTN is a distinct component located at the caudal part of the lateral hypothalamic area^13^. Functional manipulation of defined pathways has demonstrated that the PSTN is involved in motivation, the cardiovascular system, fear-induced thermoregulation, and metabolic homeostasis (by control of feeding behavior)^14–17^. Most recently, the PSTN is reported to play a key role in appetite suppression^18,19^. Meanwhile, the PSTN is implicated in conditioned taste aversion, place avoidance, and impulsive action^13,16,20^. Interestingly, PSTN neurons notably respond to arousing stimuli, especially conspicuous stimuli, mobilized by predation, and novelty reliant on the exploration, which operates on the basis of wakefulness^13,21^. However, whether the PSTN is involved in controlling wakefulness related to exploration is unknown.

Researchers have implemented single-nuclei RNA sequencing of the mouse PSTN followed by histological analysis of 19 candidate genes^22^. As a result, Calb2 (calretinin, CR) mRNA was found at high levels throughout the PSTN^22^. Here, we used fiber photometry to investigate the activity of PSTN^CR^ neurons during the spontaneous sleep-wake cycle and behavior. Chemogenetic and optogenetic methods combined with polysomnographic recordings and behavior tests were used to investigate the necessity of PSTN^CR^ neurons in arousal associated with exploratory behavior. Tracing and electrophysiology combined with optogenetic approaches were used to assess the functional connectivity between PSTN^CR^ neurons and neurons in the VTA and PB. Collectively, our results provide several lines of evidence regarding PSTN^CR^ neurons for the induction and maintenance of the awake state associated with exploration.

## Results

### Population activities of PSTN calretinin neurons increase during wakefulness and exploratory behavior

To explore the proportion of CR neurons in PSTN neurons, we stained CR and NeuN (Supplementary Fig. 1a). By counting CR neurons and NeuN neurons in the PSTN, we found that CR neurons accounted for 89.98% ± 1.51% of PSTN neurons (Supplementary Fig. 1b). Next, we injected Cre-dependent human synapsin-driven AAV vectors containing excitatory modified muscarinic G protein-coupled receptors (AAV-hSyn-DIO-hM3Dq-mCherry) and AAV-hSyn-DIO-mCherry into the PB of 6 Vglut2-Cre mice, respectively (Supplementary Fig. 2a, b). Immunohistochemistry showed that intraperitoneal (i.p.) injection of the specific hM3Dq ligand agonist clozapine-N-oxide (CNO, 1 mg/kg), but not saline, could drive c-Fos expression in the PSTN neurons (Supplementary Fig. 2c, d). And most c-Fos expressing neurons co-labeled with CR (Supplementary Fig. 2e), which means that PSTN CR neurons are the main downstream of PB glutamatergic neurons and the PSTN may be an important nucleus in the regulation of wakefulness.

**Fig. 1:**
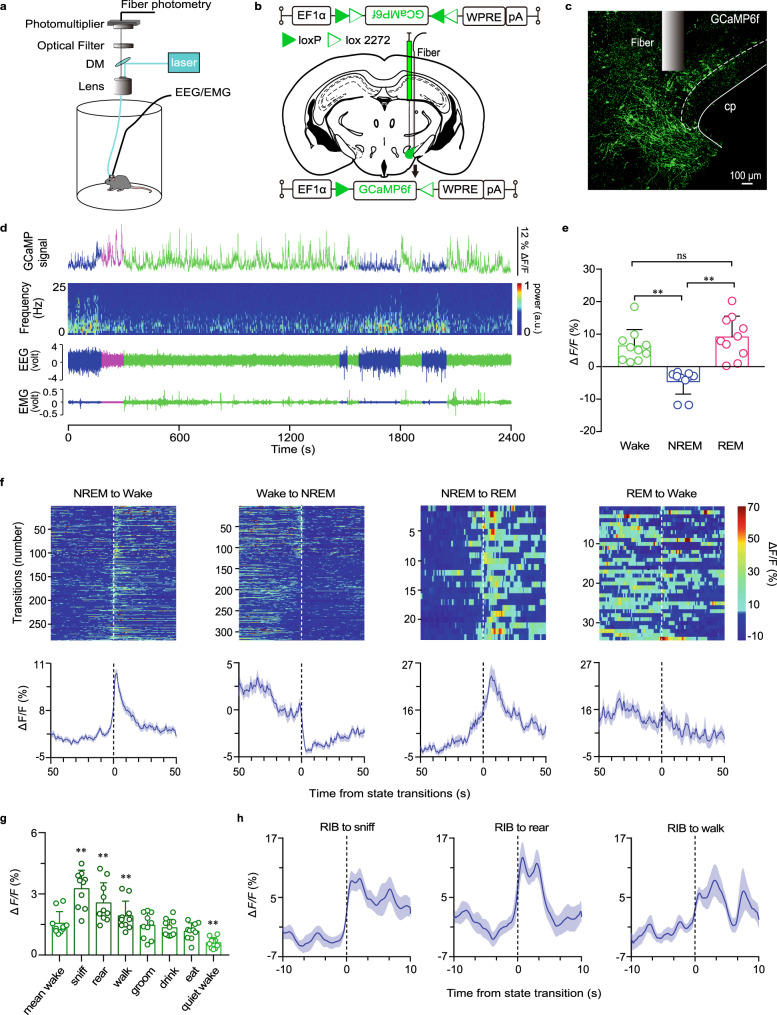
Population activity of PSTN CR neurons across sleep-wake states and sniffing, rearing, and walking behaviors. **a** Schematic of the in vivo recording configuration. **b** Diagram of unilateral viral infection area of AAV-EF1α-DIO-GCaMP6f. **c** The position of the tip of the fiber optic in the PSTN. Scale bar: 100 μm. **d** Representative fluorescence traces, EEG/EMG traces across spontaneous sleep-wake states. **e** Mean ± S.E.M. fluorescence during wake, NREM sleep, and REM sleep (n = 5, two sessions per mouse; one-way ANOVA, *F*_2,18_ = 16.34, *P* = 1.5 × 10^−4^ *P*(wake-NREM) = 7.597 × 10^−3^, (wake-REM) = 0.5851, (REM-NREM) = 3.37 × 10^−4^. ΔF/F, change in fluorescence from the median of the entire time series. **f** Fluorescence signal transformation aligned to sleep-wakefulness state transitions. Upper panel, individual transitions with color-coded fluorescence intensity (NREM to wake, *n* = 283; wake to NREM, *n* = 319; NREM to REM, *n* = 23; REM to wake, *n* = 34). Lower panel, mean (blue trace) ± S.E.M. (gray shading) showing the average calcium transients from all the transitions. **g** Fluorescence (mean ± S.E.M.) during the behavior of mean wake, sniff, rear, walk, groom, drink, eat and quiet wake (*n* = 5 mice, two sessions per mouse; one-way ANOVA, *F*_7,63_ = 18.9, *P* = 2.95 × 10^−7^ *P* = 1.13 × 10^−3^(mean wake-sniff), 3.15 × 10^−3^(mean wake-rear), 0.0228(mean wake-walk), 6.70 × 10^−3^(mean wake-quiet wake)). **h** Mean (blue trace) ± S.E.M. (gray shading) showing the average calcium transients from relatively inactive behaviors (RIB) to sniff, rear, or walk state (transition to sniff, *n* = 15; transition to rear, *n* = 13; transition to walk, *n* = 10). *P < 0.05, **P < 0.01.

To assess the real-time population activity of PSTN CR neurons during sleep and wakefulness in freely moving mice, we infected the AAV encoding the fluorescent calcium indicator GCaMP6f (AAV-EF1α-DIO-GCaMP6f) in the PSTN of CR-Cre mice. We next implanted an EEG/EMG electrode and optical fiber on the skull to record the EEG/EMG and calcium signals of PSTN CR neurons, and used a camera to observe the mouse activity simultaneously (Fig. 1a–c). By recording the EEG/EMG and calcium signal, we found that the activity of the PSTN CR neurons changed along with sleep-wake stage transformation (Fig. 1d–f); i.e., the GCaMP6f signal of PSTN CR neurons was higher during wakefulness or rapid eye movement (REM) sleep than non-REM (NREM) sleep (Fig. 1e, f, Supplementary Fig. 3). Meanwhile, the GCaMP signal of the PSTN CR neurons changed to be higher before the transition from NREM sleep to wakefulness and lower before the transition from wakefulness to NREM sleep (Fig. 1f, Supplementary Fig. 3). Interestingly, by analyzing the mouse behavior and calcium signals of PSTN CR neurons, we found that the PSTN CR neuron population activity increased in exploratory behaviors such as sniffing, rearing, and walking during mean wake, but decreased during quiet wake (Fig. 1g). The GCaMP signal of the PSTN CR neurons increased before the transition to the state of sniffing, rearing, and walking (Fig. 1h) compared to relatively inactive behaviors (RIB). These results indicate that the population activity of PSTN CR neurons is associated with wakefulness and exploration.

### Chemogenetic activation of PSTN CR neurons is sufficient to increase wakefulness associated with exploration

**Fig. 2:**
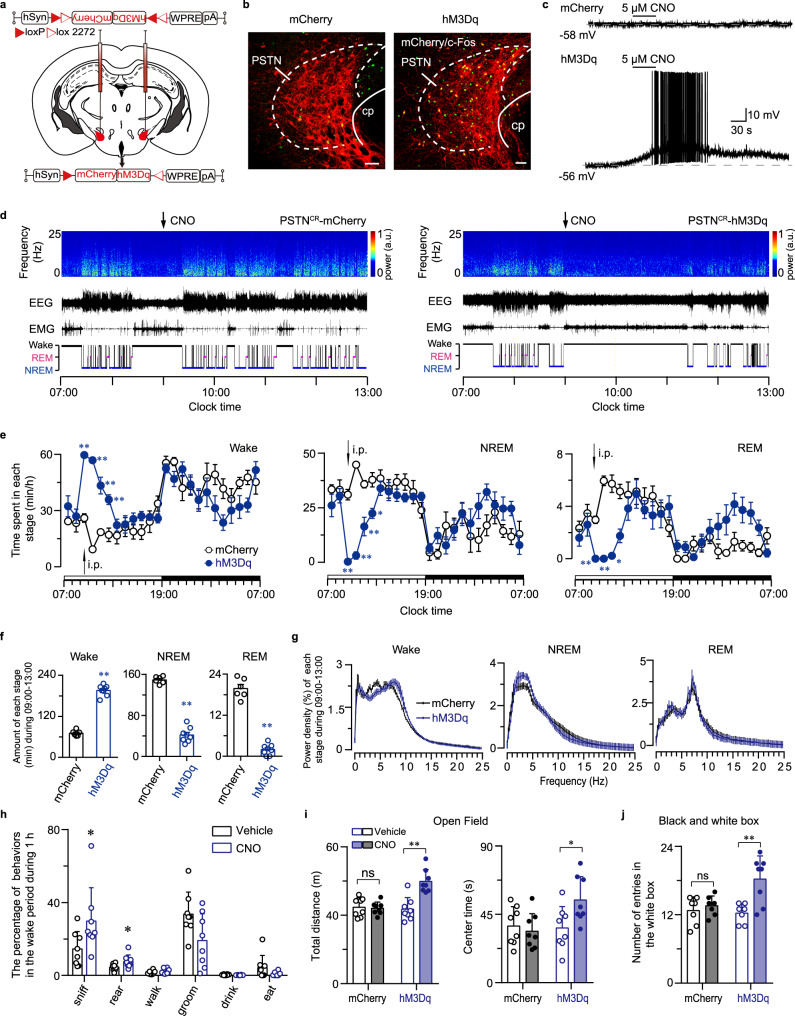
Chemogenetic activation of PSTN CR neurons increased wakefulness and exploratory behaviors. **a** Diagram of the bilateral viral infection area of AAV-hSyn-DIO-hM3Dq-mCherry in the PSTN. **b** Representative photomicrographs of the PSTN depicting mCherry (red), c-Fos (green), and merge (yellow) images of PSTN-mCherry and PSTN-hM3Dq mice after administration of CNO. PSTN: parasubthalamic nucleus, cp: cerebral peduncle. Scale bar: 50 µm. **c** Representative voltage traces recorded from an mCherry-expressing neuron and an hM3Dq-expressing neuron during the application of CNO, which produced depolarization and firing in a patched hM3Dq-expressing neuron. **d** Examples of relative EEG power, EEG/EMG traces, and hypnograms over 6 h following CNO injection of PSTN-mCherry or PSTN-hM3Dq mice at 09:00. **e** Time course changes in wakefulness, NREM sleep, and REM sleep after administration of CNO to PSTN-mCherry or PSTN-hM3Dq mice (mCherry mice: *n* = 6, hM3Dq mice: *n* = 8; Two-way ANOVA, Wake: *F*_1,12_ = 76.13, *P* = 1.53 × 10^−6^; NREM: *F*_1,12_ = 78.02, *P* = 1.34 × 10^−6^; REM: *F*_1,12_ = 8.66, *P* = 0.0123). **f** Total time spent in each stage for 4 h after CNO injection of PSTN-mCherry or PSTN-hM3Dq mice. (mCherry mice: *n* = 6, hM3Dq mice: *n* = 8; unpaired two-sided *t-test*, Wake: *t*_12_ = 17.76, *P* = 5.548 × 10^−10^; NREM: *t*_12_ = 16.69, *P* = 1.142 × 10^−9^ REM: *t*_12_ = 14.8, *P* = 4.542 × 10^−9^). **g** EEG power density of wakefulness, NREM sleep, and REM sleep during the 4 h after CNO injection of PSTN-mCherry or PSTN-hM3Dq mice (mCherry mice: *n* = 6, hM3Dq mice: *n* = 8; Two-way ANOVA; not statistically significant). **h** Quantification of the behaviors observed in mice in the 1 h following CNO or saline injection at 21:00 (*n* = 8, paired two-sided *t-test*; sniff: *t*_7_ = 3.041, *P* = 0.0188; rear: *t*_7_ = 2.448, *P* = 0.0443). Bars represent the mean percentage of awaken time (±SEM) that mice spent conducting each behavior. **i**, **j** Chemogenetic activation of PSTN CR neurons increased the locomotion and center time in the open-field test (*n* = 8, unpaired two-sided *t-test*; total distance: *t*_7_ = 4.806, *P* = 0.0003; center time: *t*_7_ = 2.502, *P* = 0.0254) (**i**) and the number of entries in the white box (mCherry groups and hM3Dq+vehicle: *n* = 7, hM3Dq+CNO: *n* = 8; unpaired two-sided *t-test*, *t*_13_ = 3.604, *P* = 0.0032) (**j**). Data represent mean ± SEM, *P < 0.05, **P < 0.01.

To examine the effect of PSTN CR neuron in sleep-wake regulation and exploration, we bilaterally injected AAV-hSyn-DIO-hM3Dq-mCherry and AAV-hSyn-DIO-mCherry into the PSTN of CR-Cre mice (PSTN-hM3Dq mice and PSTN-mCherry mice; Fig. 2a, Supplementary Fig. 4a, b). Within the PSTN of CR-Cre mice, cell-surface expression of hM3Dq receptors in CR neurons was visualized via red fluorescent mCherry protein. Immunohistochemistry showed that i.p. injection of CNO (1 mg/kg) could drive c-Fos expression in hM3Dq-expressing neurons in the PSTN compared with control group (Fig. 2b). Whole-cell current-clamp recordings showed that CNO treatment induced depolarization in hM3Dq/mCherry-positive PSTN^CR^ neurons, not in only mCherry-positive PSTN^CR^ neurons (Fig. 2c). Thus, the chemogenetic system used in this study activated PSTN CR neurons both in vivo and in vitro.

To monitor sleep-wake behavior, EEG/EMG electrodes were implanted in PSTN-mCherry and PSTN-hM3Dq mice. CNO (1 mg/kg) were administered i.p. into both group mice at 09:00 (2 h after lights on), respectively. Compared to control group, CNO injection in PSTN-hM3Dq mice at 9 a.m. produced 1 h of continuous wakefulness and significantly increased wakefulness for 4 h after administration (Fig. 2d, e). Regarding the total amount of time spent in wakefulness, NREM sleep, and REM sleep during the 4 h post-injection period, CNO resulted in a 176.21% increase in wakefulness, with 71.81% and 90.08% reductions in NREM and REM sleep of PSTN-hM3Dq mice, respectively (Fig. 2f). The EEG power spectra of each behavioral state (wake, NREM and REM sleep) following CNO-induced wakefulness were similar to those displayed during spontaneous sleep- wakefulness (Fig. 2g). Meanwhile, in order to explore the role of PSTN CR neurons on sleep-wake behavior in dark phase, we administered CNO to both PSTN-mCherry and PSTN-hM3Dq mice at 21:00 (Supplementary Fig. 5). The results showed that activation of PSTN CR neurons in dark phase induced continuous wakefulness for 4 h, illustrating the strong effect of PSTN CR neurons in the maintenance of wakefulness. On the whole, these results indicate that selective activation of PSTN CR neurons is sufficient to generate and maintain wakefulness for 4 h.

To clarify the physiological significance of wakefulness induced by activating PSTN CR neurons, we used a video-based behavioral analysis using a mouse homecage behavior analysis system (HomeCageScan Version 3.00). Compared to the saline-injected mice during 21:00–22:00, the proportion of exploratory behavior, such as sniffing and rearing, was significantly higher during CNO-induced wakefulness (Fig. 2h). There was no significant difference in the percentage of walking, drinking, grooming, or eating in CNO-activated mice compared to controls (Fig. 2h). We next conducted a series of behavioral tests to further explore whether the arousal caused by PSTN activation is related to emotion. The locomotion and the time spent in the central area (center time) of mice in an open-field test (OFT) increased (Fig. 2i); meanwhile, the number of entries into the light box during the light-dark box test increased after the activation of PSTN CR neurons (Fig. 2j). These findings indicate that activation of PSTN CR neurons is sufficient to increase locomotion and exploratory behavior. In contrast, there were no significant changes in open arm time in the elevated plus-maze (EPM) test or the immobile time in the tail suspension test (Supplementary Fig. 4c, d), suggesting that activation of PSTN CR neurons did not induce anxiety- or depression-like behaviors. Above all, the wakefulness induced by PSTN CR neuron activation occurred alongside increased locomotion in the OFT and exploratory behavior. Taken together, these results indicate that wakefulness induced by activating PSTN CR neurons is associated with exploration.

### Optogenetic activation of PSTN CR neurons during NREM sleep immediately woke the mice and increased wakefulness and exploration

**Fig. 3:**
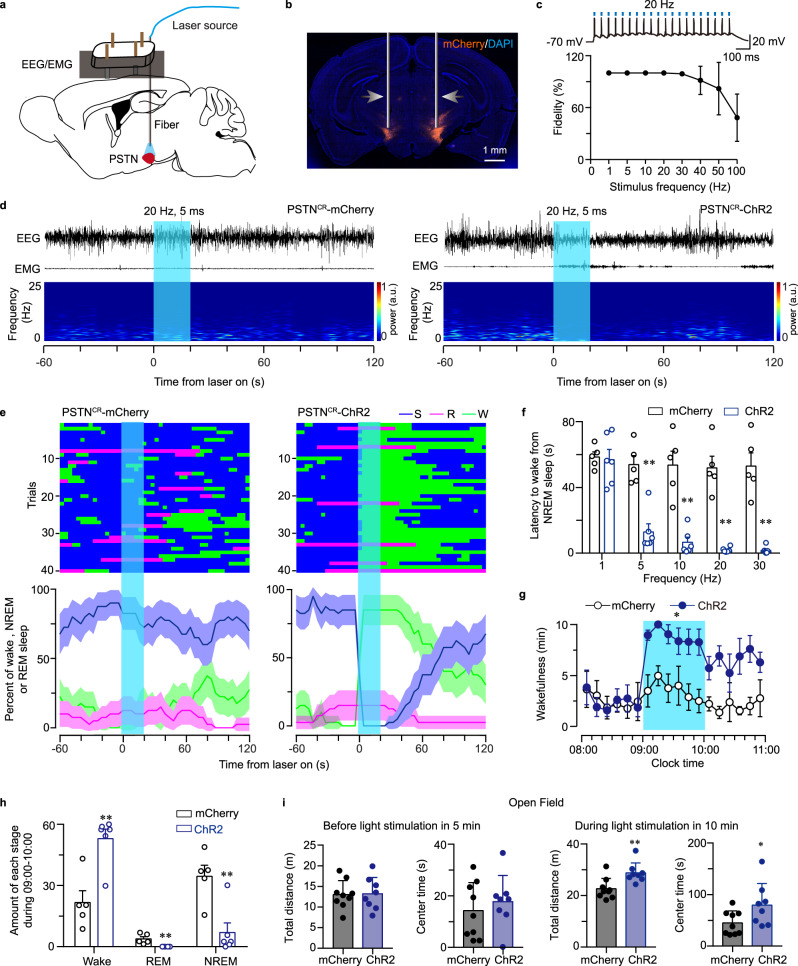
Optogenetic activation of PSTN CR neurons induces a rapid transition from NREM sleep to wakefulness and increases exploratory behaviors. **a** Schematic of the in vivo optical stimulation. **b** Representative photomicrographs of the PSTN depicting mCherry expression from a CR-Cre mouse microinjected with Cre-dependent AAV vectors containing ChR2. Scale bar: 1 mm. **c** Brief light pulses of light (5 ms, 1–100 Hz) evoked action potentials with 100% frequency fidelity in the 1, 5, 10, 20, and 30 Hz in CR neurons (*n* = 5 biologically independent cells). An example spike evoked by light pulses (20 Hz) was showed in the upside. **d** Representative EEG/EMG traces and the corresponding heatmap of EEG power spectra illustrating behavioral responses to optogenetic activation in mCherry and ChR2 mice during the 3-min recording. Blue columns indicate the photostimulation period (20 s). **e** Illustration showing the 40 NREM or REM sleep-to-wake transitions induced by photostimulation (20 Hz, 5 ms) in mCherry or ChR2 mice. Quantification was based on an average of 8 stimulations per mouse. **f** Latencies of transitions from NREM sleep to wakefulness after photostimulation at different frequencies (mCherry: *n* = 5, ChR2: *n* = 6, unpaired two-sided *t-test*, 1 Hz: *t*_9_ = 0.2204, *P* = 0.8305; 5 Hz: *t*_9_ = 5.594, *P* = 3.37 × 10^−4^; 10 Hz: *t*_9_ = 5.583, *P* = 3.42 × 10^−4^; 20 Hz: *t*_9_ = 7.83, *P* = 2.63 × 10^−5^; 30 Hz: *t*_9_ = 6.938, *P* = 6.77 × 10^−5^). **g** Time course of wakefulness during the semi-chronic optogenetic experiment (20 Hz/5 ms, 30 s on/30 s off). The column indicates the photostimulation period (1 h) of mCherry and ChR2 groups (mCherry: *n* = 5, ChR2: *n* = 6, Two-way repeated-measures ANOVA; *F*_1,9_ = 14.31, *P* = 4.33 × 10^−3^). **h** Total amounts of each stage in mCherry and ChR2 groups (mCherry: *n* = 5, ChR2: *n* = 6, unpaired two-sided *t-test*; Wake: *t*_9_ = 4.25, *P* = 2.142 × 10^−3^; REM: *t*_9_ = 3.819, *P* = 3.664 × 10^−3^; NREM: *t*_9_ = 3.862, *P* = 3.835 × 10^−3^). **i** There was no significant difference between the mCherry group and the ChR2 group in the locomotion and center time in the OF test before light on (left panel). The locomotion and center time were significantly increased in the ChR2 group compared to the mCherry group after light on (right panel, mCherry: *n* = 9, ChR2: *n* = 8; unpaired two-sided *t-test*; Total distance: *t*_15_ = 3.238, *P* = 0.0055; Center time: *t*_15_ = 2.147, *P* = 0.0486). Data represent mean ± SEM, *P < 0.05, **P < 0.01.

To explore the temporal properties of arousal responses evoked by activating PSTN CR neurons, we delivered AAV carrying Cre-dependent optogenetic stimulation of channelrhodopsin-2 (AAV-hEF1a-DIO-ChR2-mCherry) and AAV-hEF1a-DIO-mCherry to the PSTN of CR-Cre mice (Fig. 3a, b). Whole-cell patch-clamp recordings of acutely prepared brain slices containing the PSTN from CR-Cre mice were applied to test the response of ChR2-expressing neurons to optogenetic stimulation. Brief light pulses of light (5 ms, 1–30 Hz) evoked action potentials with high frequency fidelity in ChR2-expressing CR neurons (Fig. 3c). We then stimulated the PSTN in vivo in PSTN-ChR2 mice with 5-ms pulses of blue light in the frequency range of 1–30 Hz for 20 s when the mice were falling into NREM or REM sleep. During REM sleep, photostimulation of the PSTN CR neurons in PSTN-ChR2 mice had no significant effect on REM duration. Transient photostimulation at 20 Hz for 20 s of the bilateral PSTN produced immediate transitions from NREM sleep to wakefulness in PSTN-ChR2 mice, but not in PSTN-mCherry mice (Fig. 3d, e). Photostimulation at 1–30 Hz for 20 s during NREM sleep during the light (inactive) period showed that the latency to wakefulness from NREM sleep decreased along with increasing frequency after the onset of photostimulation in PSTN-ChR2 mice (Fig. 3f). Sustained activation of PSTN CR neurons in PSTN-ChR2 mice with a semi-chronic optical stimulation procedure (5 ms blue light pulses at 20 Hz for 30 s, every 1 min for 1 h) induced profound arousal, resulting in an increase in wakefulness for 144.90% during the light period between 9:00 and 10:00, compared with PSTN-mCherry mice (Fig. 3g, h). Meanwhile, the amount of time spent in both NREM sleep and REM sleep was significantly reduced (Fig. 3h). Taken together, these findings demonstrate that PSTN CR neurons are potent in initiating and maintaining wakefulness.

Exploration and locomotion can be reflected by the time in the central area and total distance in the OFT, respectively. By optical activation of PSTN CR neurons in the OFT for 10 min (5 ms blue light pulses at 20 Hz for 30 s, every 1 min for 10 min), we found that the center time of mice was prolonged and the total distance of activity increased (Fig. 3i), indicating that optogenetics-induced arousal is sufficient for exploratory behavior and locomotion during wakefulness.

### PSTN CR neurons promote wakefulness through the VTA and PB pathway

**Fig. 4:**
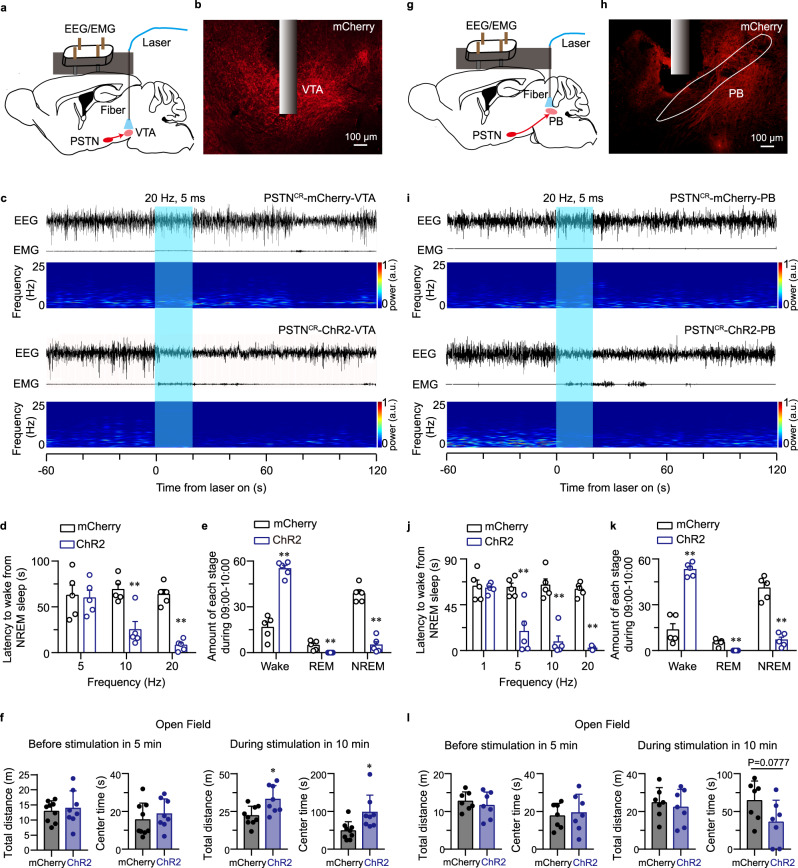
Optogenetic activation of the PSTN^CR^-VTA pathway induces a rapid transition from NREM sleep to wakefulness and increases exploratory behaviors. **a**, **g** Schematic of the optical stimulation in the VTA (**a**) and PB (**g**). **b**, **h** Representative photomicrographs of the VTA (**b**) and PB (**h**) depicting ChR2-mCherry-positive terminals (red) from the PSTN. Scale bar: 1 mm. **c**, **i** Representative EEG/EMG traces and corresponding heatmap of EEG power spectra illustrating the behavioral responses to optogenetic activation in PSTN^CR^-mCherry (up)/ChR2 (down)-VTA (**c**) and PSTN^CR^-mCherry (up)/ChR2 (down)-PB (**i**) mice during the 3 min recording. Blue columns indicate the photostimulation period (20 s). **d**, **j** Latencies of the transitions from NREM sleep to wakefulness after photostimulation of the VTA projections (*n* = 5, 5 Hz: *t*_8_ = 0.1909, *P* = 0.8533;10 Hz: *t*_8_ = 4.09, *P* = 0.0035; 20 Hz: *t*_8_ = 9.585, *P* = 1.16 × 10^−5^) (**d**) and PB projections (*n* = 5, 1 Hz: *t*_8_ = 0.2864, *P* = 0.7818; 5 Hz: *t*_8_ = 3.965, *P* = 4.15 × 10^−3^; 10 Hz: *t*_8_ = 6.534, *P* = 1.81 × 10^−4^; 20 Hz: *t*_8_ = 19.01, *P* = 6.08 × 10^−8^) (**j**) from the PSTN at different frequencies. **e**, **k** Total amounts of each stage in mCherry and ChR2 group after photostimulation of the PSTN-VTA (*n* = 5, Wake: *t*_8_ = 9.366, *P* = 1.38×10^−5^; REM: *t*_8_ = 3.496, *P* = 0.0081; NREM: *t*_8_ = 9.893, *P* = 9.2 × 10^−6^) (**e**) and PSTN-PB (*n* = 5, Wake: *t*_8_ = 8.748, *P* = 2.28 × 10;^−5^ REM: *t*_8_ = 5.23, *P* = 7.93 × 10^−4^; NREM: *t*_8_ = 8.716, *P* = 2.34 × 10^−5^) (**k**) circuits. **f** There was no significant difference between the mCherry group and the ChR2 group in the locomotion and center time in the OF test (left) before light on. The locomotion and center time are significantly increased in the ChR2 group during 10 min after photostimulation of the VTA (5 ms blue light pulses at 20 Hz for 30 s, every 1 min for 10 min) (mCherry group: *n* = 9, ChR2 group: *n* = 8; Total distance: *t*_15_ = 2.888, *P* = 0.0113; Center time: *t*_15_ = 2.791, *P* = 0.0137). **l** There was no significant difference between the mCherry group and the ChR2 group in the locomotion and center time in the OF test (left) before and after photostimulation of the PB, the photostimulation conditions are the same as (**f**) (mCherry or ChR2 group: *n* = 7, Total distance: *t*_12_ = 0.4907, *P* = 0.6325; Center time: *t*_12_ = 1.929, *P* = 0.0777). Unpaired two-sided *t-test* was used for statistical analysis (**d**–**f**, **j**–**l**). Data represent mean ± SEM, *P < 0.05, **P < 0.01.

To elucidate the role of downstream targets of PSTN CR neurons in the promotion of wakefulness, we injected anterograde tracing virus (AAV-hEF1a-DIO-ChR2-mCherry) to observe the main downstream nucleus related to arousal (Supplementary Fig. 6). We implanted a fiber optic probe above three output regions related to wakefulness regulation, including the VTA, PB, and PVT of CR-Cre mice following ChR2-mCherry and mCherry viral infusion into the PSTN (AAV-hEF1a-DIO-ChR2-mCherry or AAV-hEF1a-DIO-mCherry; Fig. 4a, b, g, h, and Supplementary Fig. 7a, b).

Transient photostimulation of the VTA and PB projections in PSTN-ChR2 mice during NREM sleep significantly decreased the latency to wake along with increasing frequency after the onset of photostimulation in PSTN-ChR2 mice compared with PSTN-mCherry mice (**P < 0.01 for both projections; Fig. 4c, d, i, j), while photostimulation of the PVT projections had no significant effect on NREM sleep duration (Supplementary Fig. 7c). To examine the capacity of PSTN^CR^ projections to maintain wakefulness, we delivered optical stimulation (5 ms blue light pulses at 20 Hz for 30 s, every 1 min for 1 h) during 9:00–10:00 in the light phase. Long-term stimulation of VTA and PB projections is sufficient for mice to increase wakefulness for 1 h (**P < 0.01 for both projections; Fig. 4e, k). Meanwhile, we found that optogenetic stimulation of PSTN^CR^-VTA projections, but not PSTN^CR^-PB projections with a long-term procedure (5 ms blue light pulses at 20 Hz for 30 s, every 1 min for 10 min), increased locomotion and exploratory time in the central area of the OFT in PSTN-ChR2 mice (Fig. 4f, l). These data revealed that the activation of PSTN^CR^-VTA or PSTN^CR^-PB projections was sufficient to promote arousal, while stimulating the PSTN^CR^-VTA pathway alone could increase locomotion and exploratory behavior during wakefulness. Long-term stimulation of PVT projections had no significant effect on sleep-wake amount (Supplementary Fig. 7d, e).

As VTA dopaminergic neurons play a key role in the regulation of locomotion, arousal, and motivated behavior^2,23^ and the PSTN is the major input to the VTA dopamine neurons, we investigated whether PSTN CR neurons have direct functional connections with VTA dopamine neurons. To confirm this hypothesis, we injected the AAV-hEF1a-DIO-ChR2-mCherry construct into the PSTN and the AAV-TH-EYFP-WPRE-pA construct into the VTA of CR-Cre mice (Supplementary Fig. 8a). We identified the TH-positive (TH^+^) and TH-negative (TH^−^) neurons by electrophysiological characteristics and EYFP fluorescence (Supplementary Fig. 8b–d). Then, we recorded the activity of VTA TH^+^ neurons under blue-light stimulation in acute slices in vitro. The result showed that stimulation of the terminals from the PSTN CR neurons evoked excitatory postsynaptic currents (EPSCs) in most of the TH^+^ neurons recorded in the VTA within 5 ms (Supplementary Fig. 8e, f; 82%). Moreover, we found that the light-evoked EPSCs were completely blocked by the AMPA and NMDA receptor antagonists NBQX and D-APV (Supplementary Fig. 8g), indicating that these responses were mediated by glutamate released from PSTN CR neurons. Additionally, we observed the electrophysiological characteristics of recorded VTA TH^+^ neurons connecting to the PSTN CR neurons, including the typical traces in response to current injections (Supplementary Fig. 8h) and the distribution of resting membrane potentials (Supplementary Fig. 8i). Above all, the electrophysiological results indicated that PSTN CR neurons principally targeted VTA TH^+^ cells, which putatively underlies the control of arousal and exploratory behavior by PSTN CR neurons. Meanwhile, we recorded the functional connections between PSTN and PB, and the electrophysiological results showed that optogenetic stimulation induced AMPAR/NMDA-mediated EPSCs in the PB, with dense mCherry-positive axonal terminals of PSTN CR neurons (Supplementary Fig. 9).

Taken together, our findings indicate that PSTN modulates arousal through PB and VTA, but only through VTA to regulate arousal associated with exploration.

### PSTN CR neurons are necessary for wakefulness associated with exploration

**Fig. 5:**
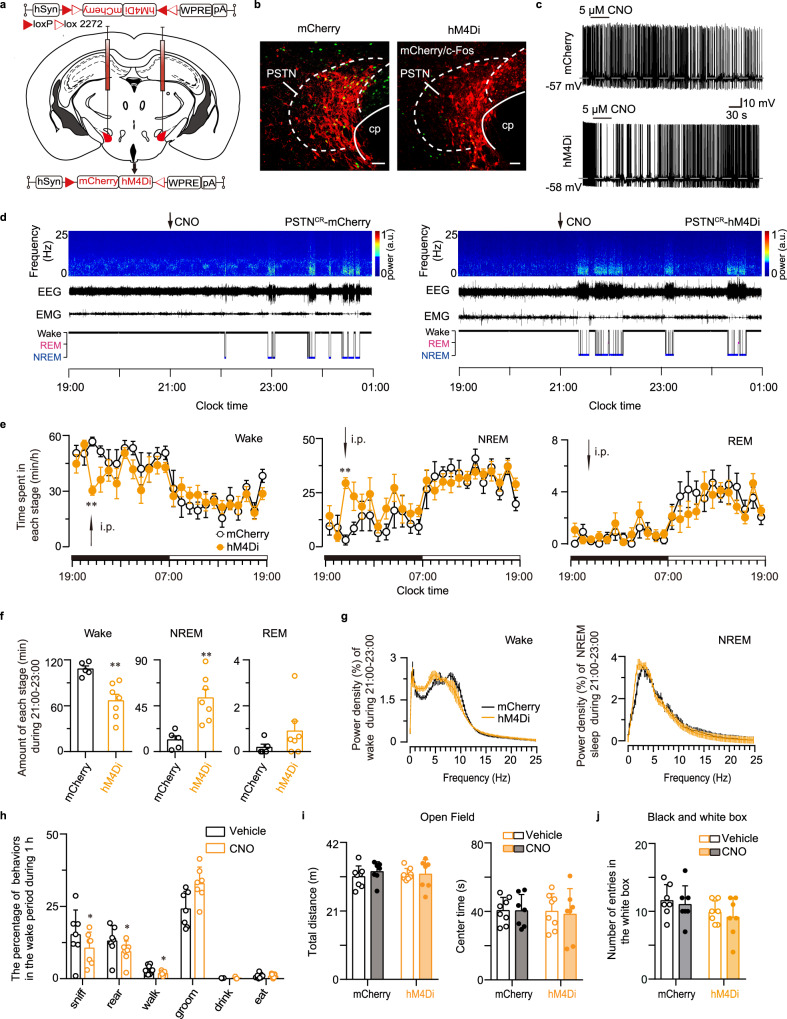
Chemogenetic inhibition of PSTN CR neurons decreases wakefulness and exploratory behaviors. **a** Diagram of bilateral viral infection area of AAV-hSyn-DIO-hM4Di-mCherry in the PSTN. **b** Representative photomicrographs of the PSTN depicting mCherry (red), c-Fos (green), and merge (yellow) images of PSTN-mCherry and PSTN-hM4Di mice after administration of CNO. CNO decreases c-Fos expression in hM4Di-expressing neurons in the PSTN. Scale bar: 50 µm. **c** Representative voltage traces recorded from an mCherry-expressing neuron and an hM4Di-expressing neuron during the application of CNO, which reduced the firing rate in a patched hM4Di-expressing neuron. **d** Examples of relative EEG power, EEG/EMG traces, and hypnograms over 4 h following CNO injection of PSTN-mCherry or PSTN-hM4Di mice at 21:00. **e** Time course changes in wakefulness, NREM sleep, and REM sleep after administration of CNO to PSTN-mCherry or PSTN-hM4Di mice (mCherry group: *n* = 5, hM4Di group: *n* = 7; Two-way ANOVA; Wake: *F*_1,10_ = 11.73, *P* = 0.0065; NREM: *F*_1,10_ = 12.53, *P* = 0.0054; REM: *F*_1,10_ = 2.22, *P* = 0.1671). **f** Total time spent in each stage for 2 h after CNO injection of PSTN-mCherry or PSTN-hM4Di mice (mCherry group: *n* = 5, hM4Di group: *n* = 7; unpaired two-sided *t-test*; Wake: *t*_10_ = 3.835, *P* = 0.0033; NREM: *t*_10_ = 3.821, *P* = 0.0034; REM: *t*_10_ = 1.326, *P* = 0.2142). **g** EEG power density of wakefulness and NREM sleep during the 2 h after CNO injection of PSTN-mCherry or PSTN-hM4Di mice (*n* = 7, Two-way ANOVA; not statistically significant). **h** Quantification of behaviors observed in mice during 1 h following 9 pm CNO injection or saline injection (*n* = 7, paired two-sided *t-test*; sniff: *t*_6_ = 3.077, *P* = 0.02176; rear: *t*_6_ = 2.853, *P* = 0.02906; walk: *t*_6_ = 3.37, *P* = 0.01504; groom: *t*_6_ = 2.335, *P* = 0.05824; drink: *t*_6_ = 1.419, *P* = 0.2056; eat: *t*_6_ = 0.08934, *P* = 0.9317). Chemogenetic inhibition of PSTN CR neurons decreased sniffing, rearing, and walking behaviors. **i**, **j** There was no significant change in behavior in the OFT and light-dark box test after inhibition of PSTN CR neurons (*n* = 7, unpaired two-sided *t-test*). Data represent mean ± SEM, *P < 0.05, **P < 0.01.

To investigate whether the PSTN CR neurons play a role in the promotion of physiological wakefulness, we injected the inhibitory receptor hM4Di (AAV-hSyn-DIO-hM4Di-mCherry) and AAV-hSyn-DIO-mCherry into the PSTN of CR-Cre mice (Fig. 5a, Supplementary Fig. 10a, b) to determine the effects of acute inhibition of PSTN CR neurons on wakefulness. Immunohistochemistry showed that injection of the specific hM4Di ligand agonist CNO (1 mg/kg, i.p.) decreased c-Fos expression in hM4Di-expressing neurons in the PSTN (Fig. 5b, Supplementary Fig. 10c). An in vitro study showed that CNO (5 μM) substantially reduced the spontaneous firing rate of CR neurons expressing hM4Di receptors in the PSTN (Fig. 5c). Compared to controls, the administration of CNO at 21:00 (2 h after light off) resulted in a cumulative reduction in wake of 38.58%, and a concomitant increase of 353.69% and 368.75% in NREM and REM sleep over the 2 h post injection period, respectively (Fig. 5d, e, f). Additionally, inhibition of PSTN CR neurons did not change the EEG power of wakefulness and NREM sleep (Fig. 5g). To explore the effect on sleep-wake behavior after optogenetic inhibition of PSTN^CR^ neurons, we delivered AAV-hEF1a-DIO-NphR-eYFP and AAV-hEF1a-DIO-eYFP to the PSTN of CR-Cre mice (Supplementary Fig. 11a–c). The results showed a weak trend that photoinhibition of PSTN CR neurons (constant yellow photostimulation for 1 min) slightly reduced the probability of transitions and prolonged the latency from NREM sleep to wakefulness, while there is no statistical significance (Supplementary Fig. 11d–g). Meanwhile, we lesioned the PSTN CR neurons by injecting the AAV-CAG-DIO-taCaspase3-TEVp into the PSTN (Supplementary Fig. 12a, b). Compared to vehicle group, lesion of PSTN CR neurons decreased wakefulness and increased NREM and REM sleep in both dark and light phase (Supplementary Fig. 12c, d). Taken together, these results demonstrate the necessity of PSTN CR neurons for the maintenance of physiological arousal.

To further explore whether inhibiting PSTN CR neurons is necessary for exploratory behavior during wakefulness, we employed video-based behavioral analysis as those behavioral tests during the inhibition of PSTN CR neurons (Fig. 5h–j, Supplementary Fig. 10d, e). Compared to physiological wakefulness, inhibition of PSTN CR neurons reduced the proportion of exploratory behaviors, such as sniffing, rearing, and walking (Fig. 5h). Although there was no significant change in behavior in the OFT, light-dark box test, EPM test, and TST, indicating that inhibition of PSTN CR neurons is unrelated to emotion (Fig. 5i, j, and Supplementary Fig. 10d, e). Taken together, these results indicate that PSTN CR neurons are necessary for wakefulness associated with exploration.

## Discussion

The PSTN is a distinct component of the LHA and related to various behaviors, including motivation, impulsive action, feeding, and hunting^14^. These behaviors are crucial for survival, and animals will demonstrate a high level of wakefulness to initiate or maintain these behaviors. Here, we demonstrate that PSTN CR neurons modulate wakefulness mainly through projections to the VTA and PB (Supplementary Fig. 13). To clarify the physiological role of wakefulness produced by PSTN CR neurons, we conducted behavioral analysis and found that wakefulness promoted by PSTN CR neurons and the PSTN^CR^-VTA pathway is associated with exploration. In contrast, our results indicate that the arousal induced by PSTN^CR^ neuron activation is not associated with negative emotions, including anxiety and depression, in mice. In the absence of PSTN neuron activity (i.e., by chemogenetic inhibition of PSTN CR neurons), both the amount of wakefulness and the proportion of exploratory behaviors (sniff, rear, walk) are reduced. Our findings identify a key nucleus in the regulation of wakefulness associated with exploration.

By anterograde tracing experiment, we found that PSTN CR neurons connect with several nuclei that regulated arousal including the basal forebrain (BF), VTA, PVT and PB, etc. The above results show that the PSTN is closely related to arousal regulatory nuclei morphologically. Given the close connection between PSTN CR neurons and arousal nuclei, we believe that PSTN may play a key role in the regulation of arousal. In our study, we found that PSTN CR neurons regulate wakefulness mainly through the VTA and PB. Both the VTA and PB are classical wakefulness-regulating nuclei^2,7,8,23^, and they have mutual projections with PSTN CR neurons. We speculate that PSTN CR neurons form positive feedback with both the VTA and PB when they play a role in promoting wakefulness, which is worth further study.

Exploratory behavior plays a fundamental role in the motivation, hunting, feeding, and well-being of organisms^24^, all of which demand a high level of wakefulness. Deviations in wakefulness and novelty exploring are characteristics of various psychiatric and neurological disorders. Thus, identifying the neural mechanisms mediating wakefulness and exploration is an important research topic. Previous study reported that the medial septum, dopamine system, and lateral hypothalamus played important roles in both arousal and exploration^24–28^. Here, we found that activation of PSTN^CR^ neurons promoted wakefulness and increased exploratory behavior, and vice versa. In our study, PSTN^CR^ neurons are proposed to be critical in the regulation of wakefulness associated with exploration. Silencing of PSTN CR neurons reduces arousal and the proportion of exploratory behaviors (sniff, rear, walk), potentially exposing animals to dangerous situations and making them more vulnerable to attack. Therefore, as the strong impairment of the wake-sleep cycle to be highly detrimental to the organism’s health and lifespan, our study suggests a pathway that may help to improve survival.

The VTA contains dopaminergic, GABAergic, and glutamatergic neurons, all of which are involved in the regulation of sleep and wakefulness^23^. As the PSTN sends strong monosynaptic inputs to VTA dopamine neurons^3^ and the VTA dopamine neurons play pivotal roles in regulating motivated behavior and behavioral arousal^2,29–31^, it is reasonable to assume that the PSTN CR neurons promote exploration-related arousal through VTA dopaminergic neurons. As the functional excitatory connections between PSTN CR and VTA dopamine neurons exist, which further suggest the potentially pivotal role of VTA dopamine neurons in the functions of PSTN CR neurons. We will further explore the role of VTA dopamine neurons in PSTN CR neurons to modulate arousal associated with exploration in the future research. In this study, we find that optogenetic activation of the PSTN^CR^-VTA pathway is sufficient to initiate arousal and increase the exploratory behavior in the OFT, suggesting that the PSTN^CR^-VTA pathway plays a key role in the regulation of exploration-related wakefulness.

The PB potently drives cortical arousal and wake behavior^8,9,32^. Our results show that wake-promoting PSTN CR neurons project heavily to the PB and vice versa^4,5,33^. Additionally, PSTN CR neurons form direct excitatory connections with PB neurons. The PB contains multiple neuron types and has been recognized as a sensory relay relevant to taste, pain, and multiple aspects of autonomous activities, including respiration, blood pressure, water balance, and thermoregulation^34^. Moreover, the PB is also associated with negative emotions, including aversion, fear, anxiety, and depression^35–37^, all of which suppress exploratory behavior. While optogenetic activation of the PSTN^CR^-PB pathway induces immediate and long-lasting wakefulness, PSTN^CR^-PB pathway activation is likely unrelated to exploration. As we have not clarified which type of neuron in the PB is connected with PSTN CR neurons, the physiological significance of the PSTN^CR^-PB pathway in regulating wakefulness remains to be further explored.

As PSTN CR neurons has collateral connections with multiple brain areas, including the PB, BF and central amygdala, etc. Arousal-promoting or other behavioral effects through activation of PSTN-PB or PSTN-VTA pathways, possibly are due to activation of other PSTN downstream targets collateral with the PB or VTA. This potential confounding effect needs further investigation in future.

The PSTN also sends substantial projections to PVT glutamate neurons, and stimulation of PSTN glutamatergic neuron terminals in the PVT inhibits food intake^5,6,38^. Recently, the PVT has been reported as a key wakefulness-controlling nucleus in the thalamus^10–12^. However, our antegrade tracing experiment shows that PSTN CR neurons do not have strong projections to the PVT, and that stimulation of PSTN CR neurons terminals in the PVT does not induce arousal and maintain wakefulness. However, we cannot exclude the possibility that other types of PSTN neurons projecting to the PVT regulate arousal.

In this study, we paid attentions to the role of PSTN CR neurons in wakefulness regulation. Actually, the population activity of PSTN CR neurons increased during REM sleep as shown in Fig.1e. However, neither chemogenetic nor optogenetic stimulation of PSTN CR neurons had a significant effect on REM sleep. Similar phenomena also appeared in previous studies [2, 39, 40] and have not yet been better explained. Meanwhile, PSTN CR neurons indeed connect with several nuclei involved in regulation of both wake and REM sleep, including the BF, laterodorsal tegmentum and pedunculopontine tegmentum. It’s possible that a subset of PSTN CR neurons that project directionally to REM-related neurons are involved in the regulation of REM sleep, which need further investigation.

Glutamate stimulation of PSTN neurons elicits depressor effects on blood circulation in anesthetized rats, which might be mediated in part through activation of NTS neurons^17^. However, the cardiovascular activity of the PSTN is inconsistent with the ability of PSTN CR neurons to induce wakefulness. Owing to the use of anesthesia and the lack of cell specificity in the previous study, a possible link between PSTN CR neurons and cardiovascular activity remains an open question.

In contrast, our findings are consistent with a previous observation that activation of serotonergic terminals in the STN/PSTN from the dorsal raphe nucleus does not affect anxiety- or antidepressant-like behavior in the elevated plus maze or forced swim test, respectively^20^. Recently, the posterior subthalamic nucleus, including the PSTN, was reported to mediate innate fear-associated hypothermia in mice^16^. Chemogenetic inhibition of glutamatergic neurons in the posterior subthalamic nucleus attenuates 2-methyl-2-thiazoline-evoked hypothermia^16^, indicating that the thermoregulation activity of the PSTN is consistent with its ability to induce wakefulness.

The PSTN is also connected to other nuclei with important physiological functions, including the basal forebrain, the bed nucleus of the stria terminalis, and the central amygdala^5,39–44^, suggesting that the PSTN, including the PSTN CR population, has other physiological functions. Here, we found that neither activating nor inhibiting the PSTN affects the anxiety- and depression-like behaviors of animals in the absence of modeling. We cannot fully confirm that PSTN participates in the regulation of anxiety- and depression-like behaviors under the conditions of modeling because it has a morphological connection with multiple emotional nuclei, and therefore requires further study.

In conclusion, our study proposes PSTN CR neurons mediate initiation and maintenance of wakefulness associated with exploration. The circuitry basis of wakefulness induced by PSTN CR neurons is composed of the PSTN^CR^-VTA and the PSTN^CR^-PB pathway, while exploration-related wakefulness is only induced via the PSTN^CR^-VTA pathway.

## Methods

### Ethics statement

All experimental protocols were approved by the Experimental Animal Ethics Committee of the School of Basic Medical Sciences, Fudan University (license identification number: 20200306-023). During all the experiments, we tried our best to minimize the pain and discomfort of animals.

### Animals

Adult male calretinin-IRES-Cre (C57BL/6) mice (8–12 weeks, 24–28 g) and Vglut2-IRES-Cre (C57BL/6) mice (8–12 weeks, 24–28 g) were used in the experiments. All of these mice were obtained from Dr. Miao He (Fudan University, Shanghai, China). Animals were housed in cages at an ambient temperature of 22 ± 0.5 °C and a relative humidity level of 60% ± 2% on an automatically controlled 12 h light/dark cycle (lights on at 07:00, illumination intensity 100 lx^45^) with free access to food and water.

### Surgery

Naive mice were anesthetized with 1.5% isoflurane in oxygen at a 0.8-LPM and placed in a stereotaxic apparatus before surgery. The animals were placed on a hot plate with a constant temperature of 30°C until they recovered from surgery. After anesthesia, the construct (AAV-hSyn-DIO-hM3Dq/hM4Di-mCherry or AAV-hEF1a-DIO-ChR2/NphR-mCherry) was delivered bilaterally into the PSTN (coordinates: AP = −2.3 mm, ML = ± 1.1 mm, DV = −5.1 mm) of calretinin-Cre mice for chemogenetic and optogenetic experiments, respectively. The construct (AAV-EF1α-DIO-GCaMP6f) was delivered unilaterally for the fiber-photometry experiment. The construct (AAV-CAG-DIO-taCaspase3-TEVp) was delivered unilaterally for the lesion experiment. The above AAV vectors were purchased from Shanghai Taitool Bioscience Limited Company. Microinjection of the virus vector was performed using a compressed air delivery system as following description^46–48^. Using the high-pressure nitrogen cylinder as the power source of gas propulsion, the Master-8 stimulator controls the Picospritzer III gas pressure propulsion system, pumps the gas into the glass microinjection needle, and drives the virus microinjection into the nucleus of mice.

The animals were observed daily to ensure that they can recover well from the operation. Two weeks after injection, mice were implanted with two EEG electrodes and two flexible electromyogram (EMG) wire electrodes for polysomnographic recordings. The two EEG electrodes made of stainless-steel screws were inserted through the skull into the cortex (anteroposterior [AP] ± 1.0 mm from and mediolateral [ML] + 1.5 mm). The EMG electrodes were made of insulated stainless steel. Teflon-coated wires were placed bilaterally in both trapezius muscles. All electrodes were attached to a microconnector and fixed to the skull with dental cement^49^. For calcium signal recording, the fiber optic cannula (200-mm diameter; Newton Inc., Hangzhou, China) was placed in the PSTN: 2.3 mm posterior and 1.1 mm lateral to the bregma, 5.1 mm deep from the skull and fixed on the skull using dental cement. For optogenetic experiments, the additional two fiber optic cannulas were placed on the PSTN, VTA (AP = –4.5 mm, ML = ±1.5 mm, DV = − 4.4 mm, 10° angle to the midline from either the left or right side), PB (AP = −5.0 mm, ML = ±1.3 mm, DV = −3.6 mm), or PVT (AP = −1.0 mm, ML ± 0 mm, DV − 3.2 mm). After surgery, mice were kept individually in transparent barrels for a week to resume normal activity^50^.

### Sleep-wake recording and analysis

After a recovery period of at least 7 days from surgery, animals were placed in a recording cage and connected to the recording cable to adapt to the recording conditions for 3 days.

In chemogenetic experiments, EEG/EMG signals were recorded and the data recorded of PSTN-mCherry mice were used as the control, and the data observed of PSTN-hM3Dq or PSTN-hM4Di mice were used as the experimental group. All animals were administered with CNO (1 mg/kg, C2041, LKT Laboratories, Minneapolis, Minnesota, US) intraperitoneally at 9:00 (light period) or 21:00 (dark period). After recording, animals were perfused 90 min after CNO treatment, and then the brain was removed for immunohistochemical staining.

The EEG/EMG signal was amplified, filtered (EEG, 0.5–25 Hz; EMG, 20–200 Hz), digitized at a sampling rate of 128 Hz, and recorded using Vital Recorder software (Kissei Comtec, Nagano, Japan). When completed, sleep recordings were scored off-line automatically by 4-seconds epochs for both chemogenetic and optogenetic experiments as wakefulness, REM, and NREM sleep by SLEEPSIGN according to standard criteria^51^. Finally, defined sleep-wake stages were examined visually and corrected manually, if necessary^52^. For the normalization of power density, we derived the power density of each frequency band of sleep-wake phases, and compared the power density of each frequency band to the sum of them.

### Histology and immunohistochemistry

On completion of the calcium recording, chemogenetic, and optogenetic experiments, and after CNO administration (chemogenetic), animals were deeply anesthetized by sodium pentobarbital and transcardially perfused with 30 mL 0.1 M phosphate-buffered saline (PBS) followed by 50 mL 4% paraformaldehyde (PFA) in PBS. After removal, brains were post-fixed for 24 h in 4% PFA, and then incubated in 30% sucrose in PBS solution at 4 °C until they sank. Brains were embedded in OCT compound and then sliced into three series of 30-μm sections on a freezing microtome (CM1950, Leica, Germany).

For non-fluorescent staining of c-Fos, sections were washed in PBS containing 0.3% Triton X-100 (PBST), and incubated in primary rabbit polyclonal anti-c-Fos antibody (1:10000, ABE457, Millipore, USA) diluted in PBST for 48 h at 4 °C. For chromogenic detection of c-Fos, sections were then washed three times in PBS (5 min each time), incubated in donkey anti-rabbit biotinylated IgG (1:1000, Jackson ImmunoResearch) in PBST for 2 h at room temperature (RT), washed in PBS, and incubated with ABC complex (Vectorlab, USA) in PBST for 2 h at RT. Then, sections were washed and incubated in a solution of 3, 3′ diaminobenzidine (DAB, 0.2 mg/ml) and 0.005% H_2_O_2_ in PBS containing NiCl. For double immunostaining of c-Fos and mCherry, sections were washed again after the DAB reaction and incubated with rabbit polyclonal anti-mCherry antibody (1:8000) diluted in PBST for 24 h at 4 °C. All of the above steps, except the final step (the DAB reaction with NiCl), were repeated. Finally, the DAB reaction without NiCl is carried out.

For fluorescent detection of c-Fos, calretinin, or NeuN, sections were washed in PBST, and incubated in primary rabbit antibody: anti-c-Fos (1:10000, ABE457, Millipore, USA), anti-CR (1:2000, cat. #7697, Swant, Bellinzona, Ticino), or NeuN (1:1000, MAB377B, Millipore, USA) diluted in PBST for 48 h at 4 °C. Sections were then washed three times in PBS (5 min each time) and incubated in donkey anti-rabbit Alexa Fluor-conjugated IgG antibody (1:1000, Jackson ImmunoResearch) for 2 h at room temperature (RT). Then, sections were washed three times in PBS and mounted on slides with Fluoromout-G^TM^ (Southern Biotech).

### Cell counting

Images of sections including the PSTN were captured with a 10× objective on an Olympus microscope (VS-120, Tokyo, Japan) after immunohistochemical experiments. To delineate the boundary of PSTN, we embed the brain map of mouse including the PSTN into the brain slice through Photoshop software, and overlapped the markers cerebral peduncle (cp) and the third ventricle (3 V). According to the mouse brain map (Paxinos and Franklin, 2013), CR^+^/NeuN and NeuN-positive neurons in the range of PSTN are counted, the percentage of CR neurons of the total neurons in the PSTN was then calculated. To evaluate the activity of the PSTN when PB was activated, c-Fos-positive neurons were counted in the PSTN area, and the number of c-Fos/calretinin-positive neurons were counted in this area to determine whether the calretinin-positive neurons were the main neurons affecting sleep-wake behavior in the PSTN. Cell counting was performed on three sections—the front, middle, and back of the PSTN—using Photoshop bilaterally; the total count of the three sections was used to represent the data^53,54^. CR^+^/NeuN and NeuN-positive neurons were counted to evaluate the percentage of CR^+^ neurons of the total neurons in the PSTN. The ratio of CR^+^/NeuN and NeuN-positive neurons was then calculated.

### Behavioral tests

#### Homecage recording

To evaluate the behavioral changes of mice after administration of saline or CNO, we used HomeCage Scan Version 3.00 software to analyze mouse behavior by recording videos of mice moving freely in their homecages. We defined each behavior, including sniff (to breathe air in through the nose in order to discover or enjoy the smell of something), rear (to stand up on the hind legs), walk (to move or go somewhere, always having at least two feet on the ground), groom (to clean the fur or skin of itself), drink (to have water), and eat (to have food). Through comparison with the vehicle group, we calculated the ratio of the total time of each behavior to the time of wakefulness.

#### Open-field test (OFT)

To track locomotor activity and the motivation to explore the central area in a novel environment, mice were gently placed in the center of a 40 × 40 cm open field (OF) apparatus and the activity was recorded for 10 min. The Tracking Master V3.0 (TMV3) software was used to generate primary outcome measures, including the total distance traveled (locomotion) and center time (exploration)^52,55^.

#### Elevated plus-maze test (EPM)

The EPM was performed in a crossed maze with two closed and two open arms to assess anxiety-like behavior. Each mouse was placed in the center of the crossed maze, and the total distance traveled (locomotion) and the time spent in the open arms were measured by the video and TMV3 software for 5 min.

#### Light-dark box test

The light-dark test was performed in a box divided into a dark chamber and a light chamber. Each mouse was placed in the light side and activity was recorded for 10 min by video. The number of entries in the light chamber was recorded and analyzed^52^.

#### Tail-suspension test

The mice were suspended by their tails with their heads hanging 30 cm from the ground for 6 min. The immobility time of the last 5 min was measured by video and TMV3 software.

### Optogenetic stimulation

Before stimulation, the fiber optic cannulas were connected with a 473 nm blue laser or 589 nm yellow laser diode^49,56^. Light pulse trains were generated by a stimulator (SEN-7103, Nihon Kohden, Japan) and output by an isolator (ss-102J, Nihon Kohden). For instantaneous photostimulation, each trial was applied 20 s after a stable NREM or REM sleep event by observing the online EEG/EMG display. For prolonged photostimulation, programmed light pulse trains (5 ms pulses at 20 Hz for 30 s and at 30 s intervals for 1 h) were used from 9:00 to 10:00. The recorded EEG/EMG during the same period of PSTN-mCherry mice served as the control. Animals were perfused after receiving 1 h-prolonged photostimulation for c-Fos staining. The light intensity at the tip of the optical fiber was tested by a power meter (PM10, Coherent) before each experiment time and calibrated to emit 20–30 mW/mm^2^. For the open-field test, programmed light pulse trains (5 ms pulses at 20 Hz for 30 s and at 30 s intervals for 10 min) were used after the mice had adapted to the field environment for 5 min.

### Fiber photometry recording and analysis

We used fiber photometry^49^ to record calcium signals from the cell bodies of CR neurons in the PSTN in freely moving mice. An optical fiber (1.25 mm O.D., 0.37 numerical aperture; Newton, Shanghai) was inserted toward the PSTN 3 weeks after AAV-EF1α-DIO-GCaMP6f virus injection. The optic fibers implanted in mice were connected to the fiber photometry system to excite and record from GCaMP in real time. To excite and record fluorescence signals, the optic fiber implanted in the mouse was connected to a patch cable that received a laser beam (488 nm) sent from a laser tube (488 nm OBIS 488LS; Coherent), reflected by a dichroic mirror (MD498; Thorlabs), focused by a ×10 objective lens (Olympus), and coupled to a fiber collimation package (F240FC-A, Thorlabs). A photomultiplier tube was used to bandpass filter (MF525–39, Thorlabs) and collect (R3896, Hamamatsu) the GCaMP6 fluorescence. The photomultiplier tube current was converted to voltage signals by an amplifier (C7319, Hamamatsu) and further filtered through a low-pass filter (40 Hz cut-off; Brownlee 440). To match the EEG/EMG sampling rate of 512 Hz, the photometry voltage signals were downsampled by a Power1401 digitizer and recorded with Spike2 software (CED, Cambridge, UK). To better observe the relationship between the calcium activity of PSTN CR neurons and mouse behavior, we simultaneously made a video of the autonomous activities of mice. Here, we defined behaviors other than sniff, rear, and walk as relatively inactive behaviors (RIB). Analysis of the resulting signal was performed using a procedure written by MATLAB. The dF/F value was calculated by the formula dF/F = (F-F_0_)/F_0_. The value of F and F_0_ were recorded by Spike2 software. In the analysis of sleep-wake behavior, the baseline fluorescence was defined as the fluorescence collected when the laser irradiates the recording environment. In the analysis of different behaviors, the baseline fluorescence was defined as the fluorescence collected when the laser on the PSTN CR neurons expressing GCaMP6f of relatively quiet mice.

### In vitro electrophysiology

Three weeks after injecting Cre-independent hSyn–hM3Dq (hM4Di, ChR2)-mCherry into PSTN of CR-Cre mice, coronal slices containing the PSTN, VTA and PB of CR-Cre mice were prepared for electrophysiological experiments. Mice were anesthetized with sodium pentobarbital and perfused with ice-cold sucrose-based artificial cerebrospinal fluid (ACSF), saturated with 95% O_2_ and 5% CO_2_ (pH 7.3), and containing (in mM) the following: 213 sucrose, 2.5 KCl, 26 NaHCO3, 10 glucose, 3 MgSO4, 1.25 NaH2PO4, 2 Na-pyruvate, 0.4 ascorbic acid, and 0.1 CaCl2. Brains were removed rapidly and cut in coronal slices (300 μm) with a vibrating microtome (VT1200, Leica, Germany) in ice-cold ACSF. Next, slices containing the PSTN, VTA, and PB were transferred to normal recording ACSF (in mM): 126 NaCl, 25 glucose, 2.5 KCl, 2 CaCl2, 1.25 NaH2PO4, 26 NaHCO3, and 1.0 MgSO4. Then, slices were allowed to recover at 32 °C for 30 min and were maintained at RT for 30 min before recording.

During recording, slices were submerged in a chamber and superfused with warm (30–32 °C) ACSF (2 mL/min). Slices were visualized by the low-magnification fluorescence microscope to find the target brain area (PSTN, VTA and PB), and then locate the neurons to be recorded under the high-magnification fluorescence microscope^49^. Patch electrodes (4–6 MΩ) were filled with an internal solution containing 105 mM potassium gluconate, 30 mM KCl, 4 mM ATP-Mg, 10 mM phosphocreatine, 4 mM ATP-Mg, 0.3 mM EGTA, 0.3 mM GTP-Na, and 10 mM HEPES (pH 7.3, 285–300 mOsm). A MultiClamp 700B amplifier (Axon Instruments) was used to record cell-attached and whole-cell current-clamp and voltage-clamp (held at −70 mV) at 30–32 °C. Signals were filtered at 4 kHz and digitized at 10 kHz using a DigiData 1440 A (Axon Instruments). Data were acquired and analyzed with pClamp10.3 software (Axon Instruments).

Optical stimulation (470 nm light, 5 ms pulses, 1–100 Hz) was delivered via the objective lens from a microscope-mounted blue LED (3–5 mW, Lumen Dynamics, Canada) onto the slice. When needed, 25 μM d-(−)−2-amino-5-phosphonopentanoic acid (d-APV), 20 μM 6-nitro-7-sulphamoylbenzo(f)-quinoxaline-2,3-dione (NBQX), and 100 μM picrotoxin (PTX) were added to the ACSF to block NMDA, AMPA, and GABAA receptors, respectively. As series resistance (Rs) compensation was not used, cells with Rs changes >20% were discarded.

### Statistical analysis and Reproducibility

Data are presented as the mean ± standard error of the mean (SEM). One-way analysis of variance (ANOVA) was used for comparison between two groups of fluorescence results. Two-way ANOVA was used for comparison the durations of three stages (S, W, or R) at each hour over time. Paired two-tailed student’s *t-tests* were used for comparison between two groups of Homecage behaviors. Unpaired two-tailed student’s *t-tests* were used for comparison between two groups of quantification results of c-Fos, c-Fos/CR, or CR/NeuN immunohistochemistry analysis, latencies from NREM sleep to wakefulness, and total sleep-wake amounts, behavioral results of OFT, EPM, light-dark box test, and tail-suspension test. A two-tailed *P*-value < 0.05 was considered statistically significant. Statistical analysis was performed using Prism 7.0 software. The reproducibility of micrographic and behavior experiments of optical fiber calcium recording, chemogenetics and optogenetics are consistent with the actual number of experimental animals. The repeatability of the micrograph for statistics is consistent with the number of animals. The repeatability of anterograde tracing experiments is at least three times.

### Reporting summary

Further information on research design is available in the Nature Portfolio Reporting Summary linked to this article.

## Supplementary information

Supplementary Information

Reporting Summary

## Data Availability

The raw data generated in this study have been deposited in the Figshare database under accession code: 10.6084/m9.figshare.22259794.v1. Source data are provided with this paper.

## Source data

Source Data

## Code availability

The codes that support the data of this study have been deposited in the Figshare database: 10.6084/m9.figshare.22122179.v1.
