# Supplementary material for: Parasubthalamic calretinin neurons modulate wakefulness associated with exploration in male mice
Source: Nat Commun. 2023 Apr 24;14:2346. doi: 10.1038/s41467-023-37797-y (PMC10126000; doi:10.1038/s41467-023-37797-y)
Supplement: Supplementary file 1 — Supplementary Information [file 41467_2023_37797_MOESM1_ESM.pdf]

Supplementary figures and legends

Parasubthalamic calretinin neurons modulate wakefulness associated with exploration in male mice

Han Guo[1,2,3*], Jianbo Jiang[1*], Wei Xu[1], Mu-Tian Zhang[1], Hui Chen[1], Huan-Ying Shi[4], Lu Wang[1], Miao He[1], Michael Lazarus[5], Shan-Qun Li[2#], Zhi-Li Huang[1#], Wei-Min Qu[1#]

[1]Department of Pharmacology, School of Basic Medical Sciences; State Key Laboratory of Medical Neurobiology and MOE Frontiers Center for Brain Science, and Institutes of Brain Science, Fudan University, Shanghai 200032, China.

[2]Department of Pulmonary Medicine, Zhongshan Hospital; Fudan University, Shanghai 200032, China.

[3]Department of Pharmacy, Huadong Hospital, Fudan University, Shanghai, China.

[4]Department of Pharmacy, Huashan Hospital, Fudan University, Shanghai, China.

[5]International Institute for Integrative Sleep Medicine (WPIIIIS), University of Tsukuba, Tsukuba, Ibaraki 305-8575, Japan.

[*] These authors contributed equally to this work.

[#] These authors are Co-corresponding authors.

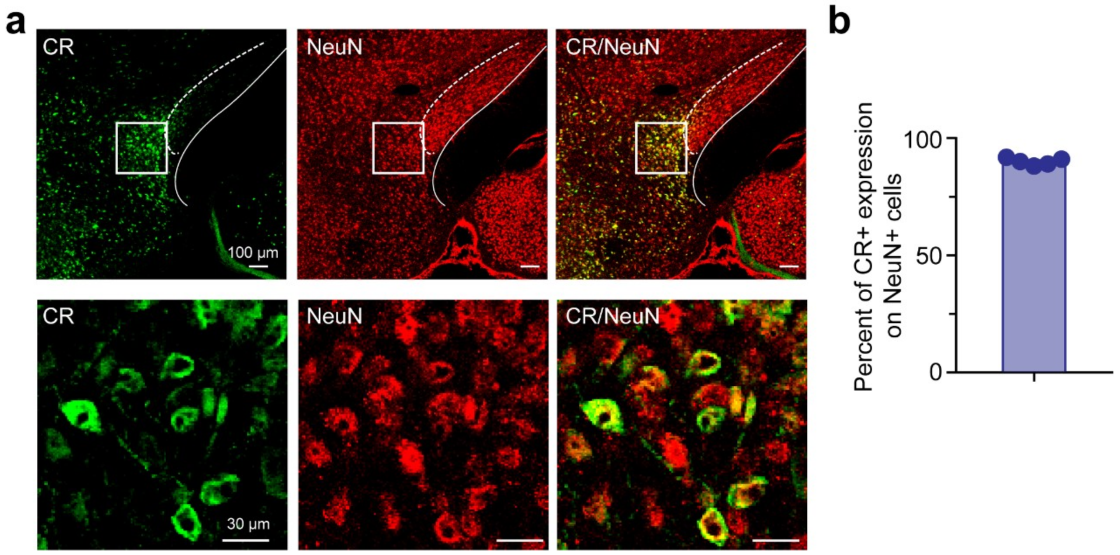

Supplementary Figure 1 Most of the PSTN neurons express CR.

a Representative photomicrographs of the PSTN depicting CR (green, left), NeuN (red, middle), and merge images (yellow, right) of a mouse. Bregma: -2.30 mm. Scale bars: 100 µm. The enlarged pictures in the box are shown under each original image. Scale bars: 30 µm. **b** The co-labeling rate of CR neurons was 89.98% ± 1.51% of the total number of neurons in the PSTN, n=5 biologically independent samples.

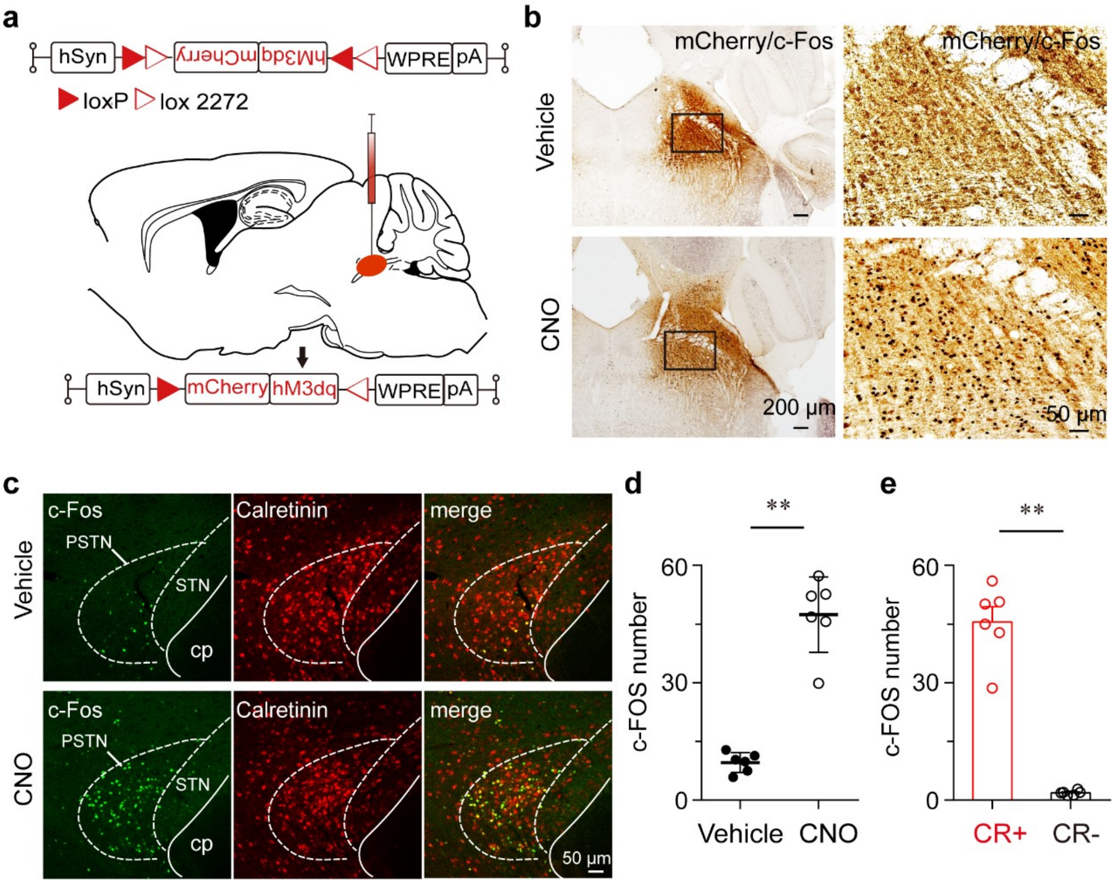

Supplementary Figure 2 Chemogenetic activation of the PB glutamatergic neurons specifically increased the c-Fos-positive neurons expressing CR in the PSTN. a Schematic diagram of the bilateral viral infection area of AAV-hSyn-DIO-hM3Dq-mCherry in the PB of Vglut2-Cre mice. **b** Representative immunohistochemical images of the PB depicting CNO-induced c-Fos (black)/mCherry (brown) co-localization. CNO drives c-Fos expression in mCherry-expressing neurons in the PB. Bregma: -5.07 mm. Scale bars: 200 μm. The enlarged drawings of the boxed regions in (**b**) are shown in the right panel. Scale bars: 50 μm. **c** Typical image of c-Fos/CR co-expressing neurons in the PSTN after CNO excitation of the PB neurons. Bregma: -2.30 mm. Scale bars: 50 μm. **d** Quantification of c-Fos staining in the PSTN after saline or CNO injection in mice (n=6, per group, unpaired two-sided *t-tests*; t_{10}=9.319, P=3.02×10^{-6}). **e** Quantification of CR staining in c-Fos-expressing neurons in the PSTN after saline or CNO injection in mice (n=6, per group, unpaired two-sided *t-tests*; t_{10}=11.27, P=5.27×10^{-7}). Data represent mean ± SEM, **P < 0.01.

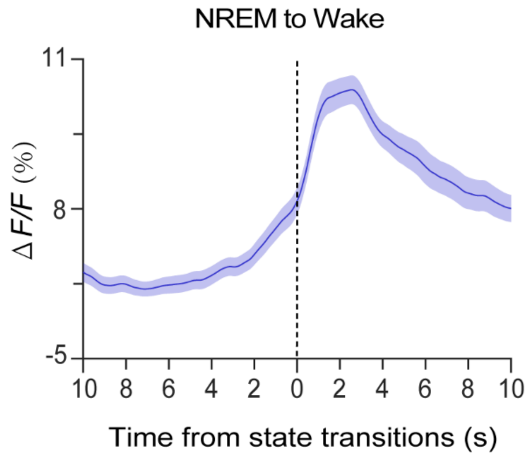

Supplementary Figure 3 The activity of PSTN CR neurons increased prior to the transition from NREM sleep to wakefulness.

Fluorescence signal transformation aligned to NREM sleep-wakefulness state transitions. mean (blue trace) ± S.E.M. (gray shading) showing the average calcium transients from all the transitions.

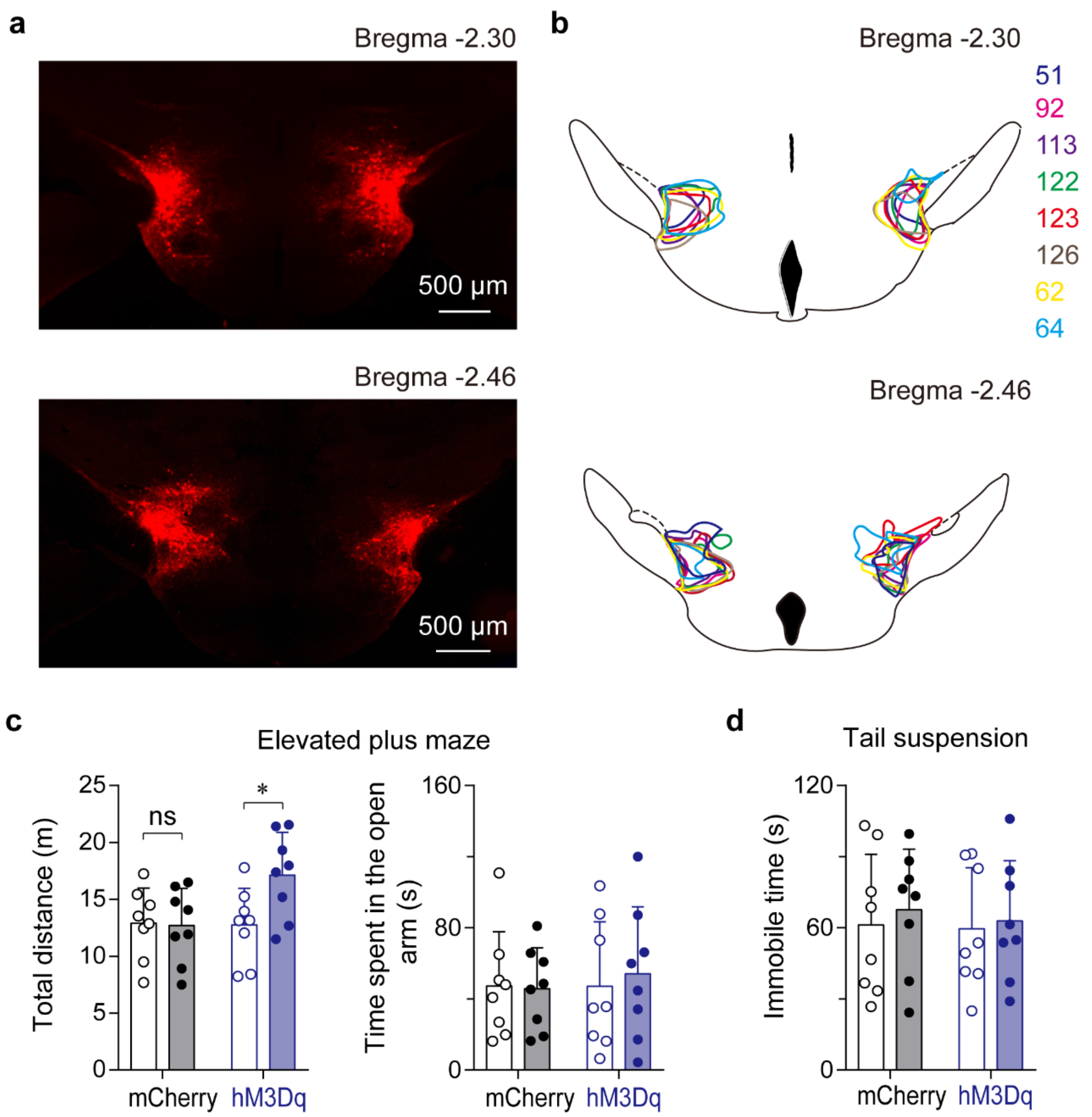

Supplementary Figure 4 Chemogenetic activation of PSTN CR neurons increased the total distance in the EPM test and didn't affect the immobile time in the TST test.

a Representative photomicrographs of the PSTN depicting mCherry (red) images of a PSTN-hM3Dq mouse (Bregma=–2.30 in the upside and Bregma=–2.46 in the downside). **b** The drawings show superimposed AAV-hSyn-DIO-hM3Dq-mCherry infection area in the PSTN of 8 mice (Bregma=–2.30 in the upside and Bregma=–2.46 in the downside). **c**, **d** Chemogenetic activation of PSTN CR neurons increased the total distance in the EPM test (n=8, unpaired two-sided *t-tests*; Total distance: t_{14}=2.506, *P*=0.0252) (**c**), and didn't change the time spent in the open arm in the EPM (**c**) and the immobile time in the TST (**d**), n=8, unpaired two-sided *t-tests*. Data represent mean ±

SEM, *P<0.05.

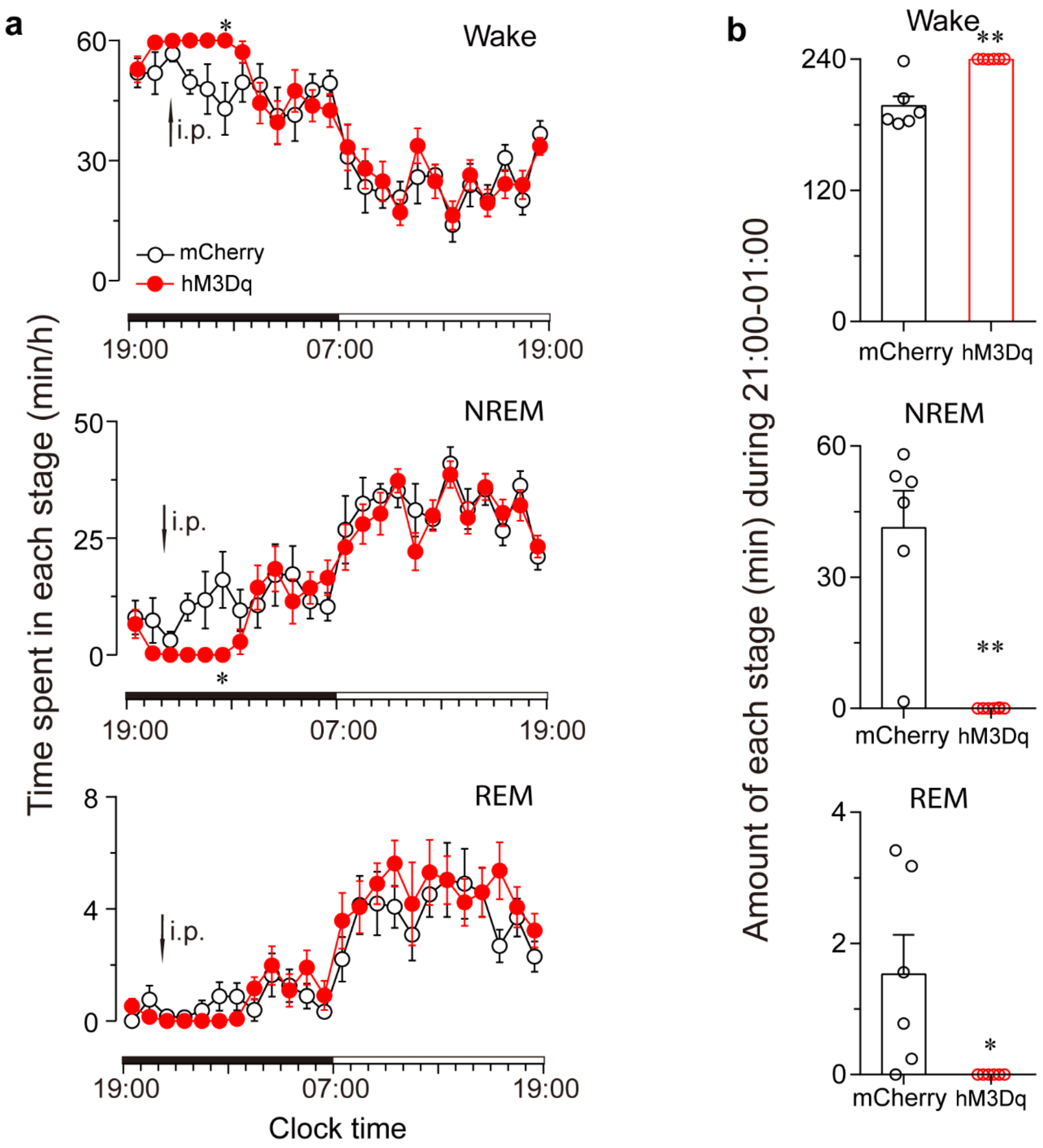

Supplementary Figure 5 Chemogenetic activation of PSTN CR neurons in dark phase induced wakefulness.

a Time course changes in wakefulness, NREM sleep, and REM sleep after administration of CNO to PSTN-mCherry and PSTN-hM3Dq mice at 21:00 (mCherry mice: n=6, hM3Dq mice: n=8; Two-way ANOVA, Wake: $F_{1,12}$=6.702, P=0.0237; NREM: $F_{1,12}$=8.4, P=0.0134; REM: $F_{1,12}$=0.0312, P=0.8627). **b** Total time spent in each stage for 4 h after CNO injection (mCherry mice: n=6, hM3Dq mice: n=8; unpaired two-sided *t-test*; Wake: t_{12}=5.651, P=1.07×10^{-4}; NREM: t_{12}=5.675,

P=1.03×10^{-4}; REM: t_{12}=2.978, P=0.01154). Data represent mean ± SEM, *P < 0.05, **P < 0.01.

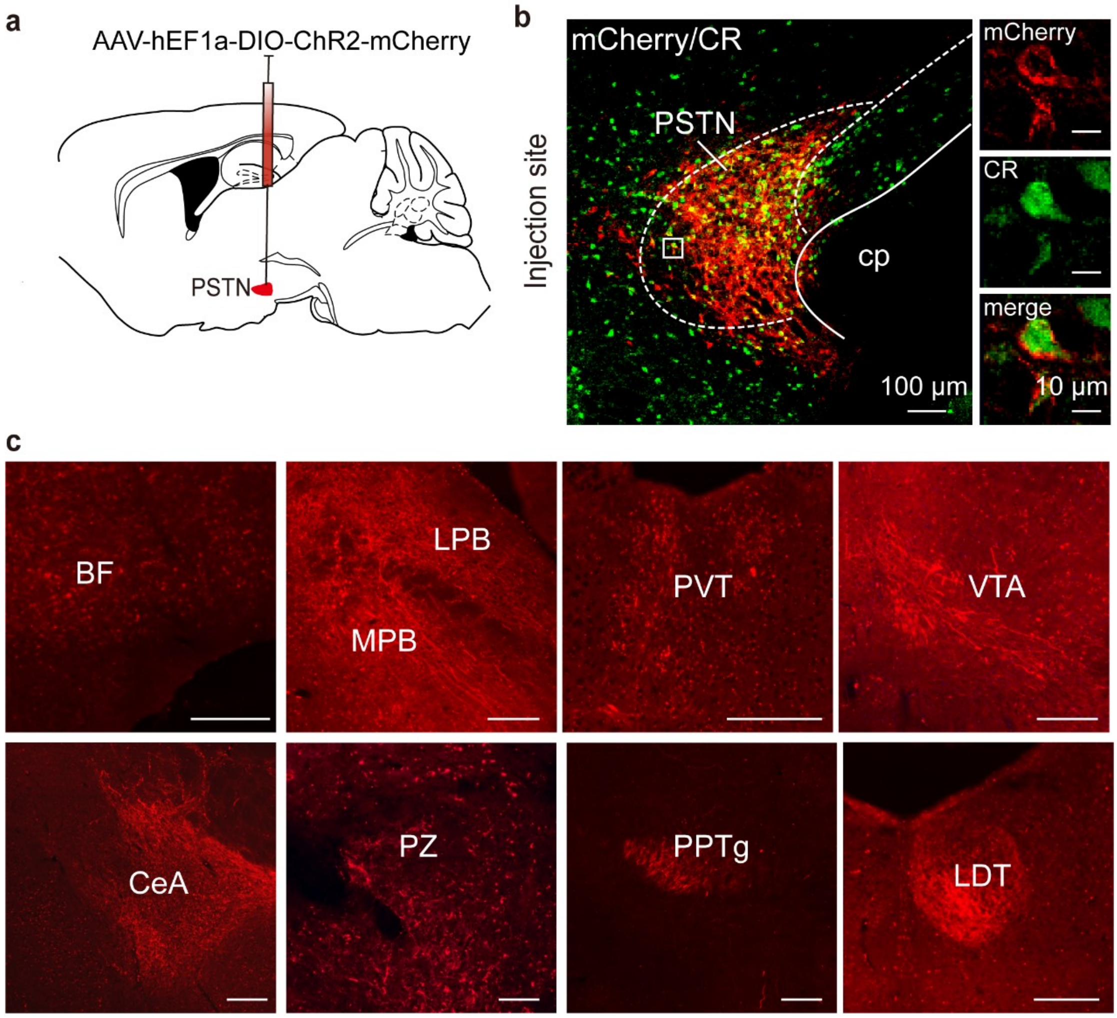

Supplementary Figure 6 PSTN CR neurons innervated the VTA, PB, and PVT.

a Schematic of the PSTN injection site and viral vectors for AAV-EF1a-DIO-ChR2-mCherry in CR-Cre mice. **b** Left, representative fluorescence image showing that almost all of the neurons infected with ChR2-mCherry co-localized with CR in the PSTN. Scale bar: 100 μm. Right, higher magnification images of the area outlined by the white box. Scale bars: 10 μm. **c** Representative image showing mCherry-labeled axons of PSTN CR neurons. BF: Basal forebrain, LPB: Lateral parabrachial nucleus, MPB: Medial parabrachial nucleus, PVT: Paraventricular thalamic nucleus, VTA: Ventral tegmental area, CeA: Central amygdala, PZ: Parafacial zone, PPTg: Pedunculopontine tegmental nucleus, LDT: Laterodorsal tegmental nucleus. Scale bar: 200 μm.

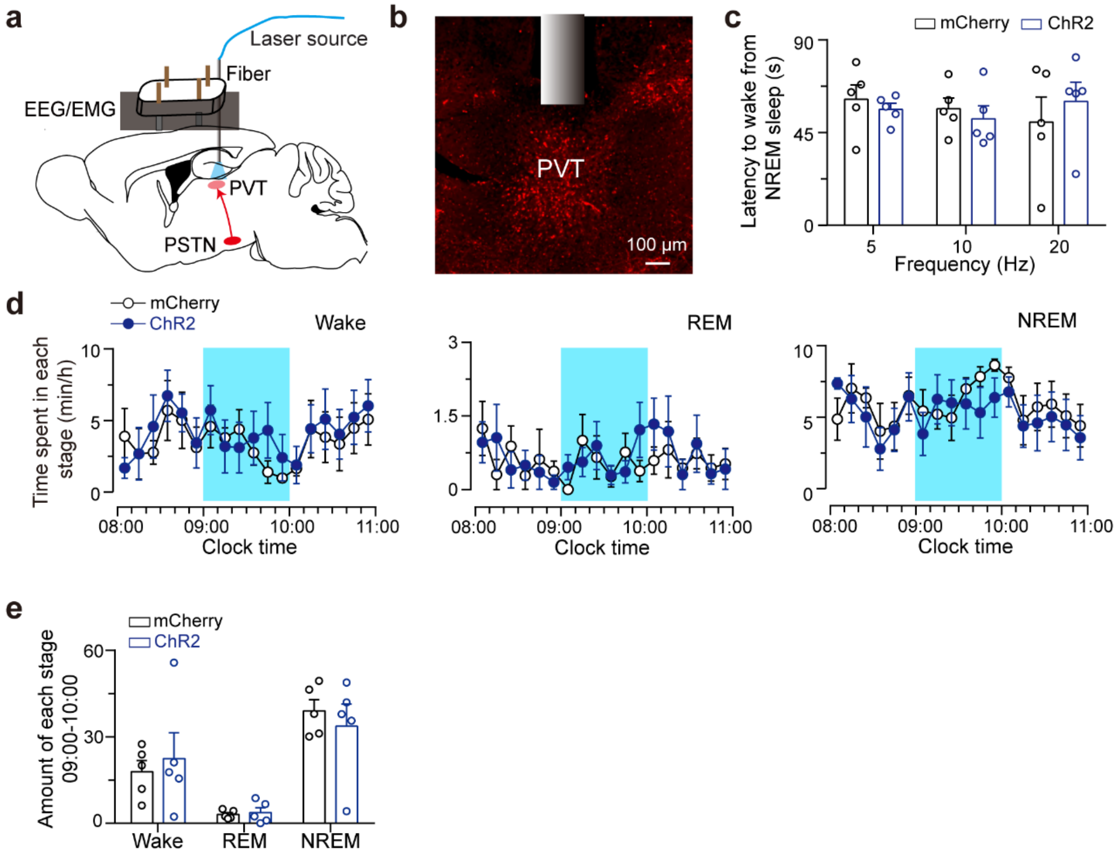

Supplementary Figure 7 Optogenetic activation of PSTN^{CR}-PVT pathway in sleeping mice did not alter sleep stages.

a Schematic of the *in vivo* optical stimulation. **b** Sections showing ChR2-mCherry-positive terminals in the PVT. Scale bar: 100 μm. **c** Latencies of transitions from NREM sleep to wakefulness after photostimulation at different frequencies (mCherry or ChR2 group: n=5, unpaired two-sided *t-test*). **d** Time course of wakefulness during the semi-chronic optogenetic experiment (20 Hz/5 ms, 30 s on/30 s off). The blue columns indicate the photostimulation period (1 h) of ChR2 group (n=5, Two-way repeated-measures ANOVA). **e** Total amounts of each stage in the baseline and photostimulation groups (n=5, unpaired two-sided *t-test*). Data represent mean ± SEM.

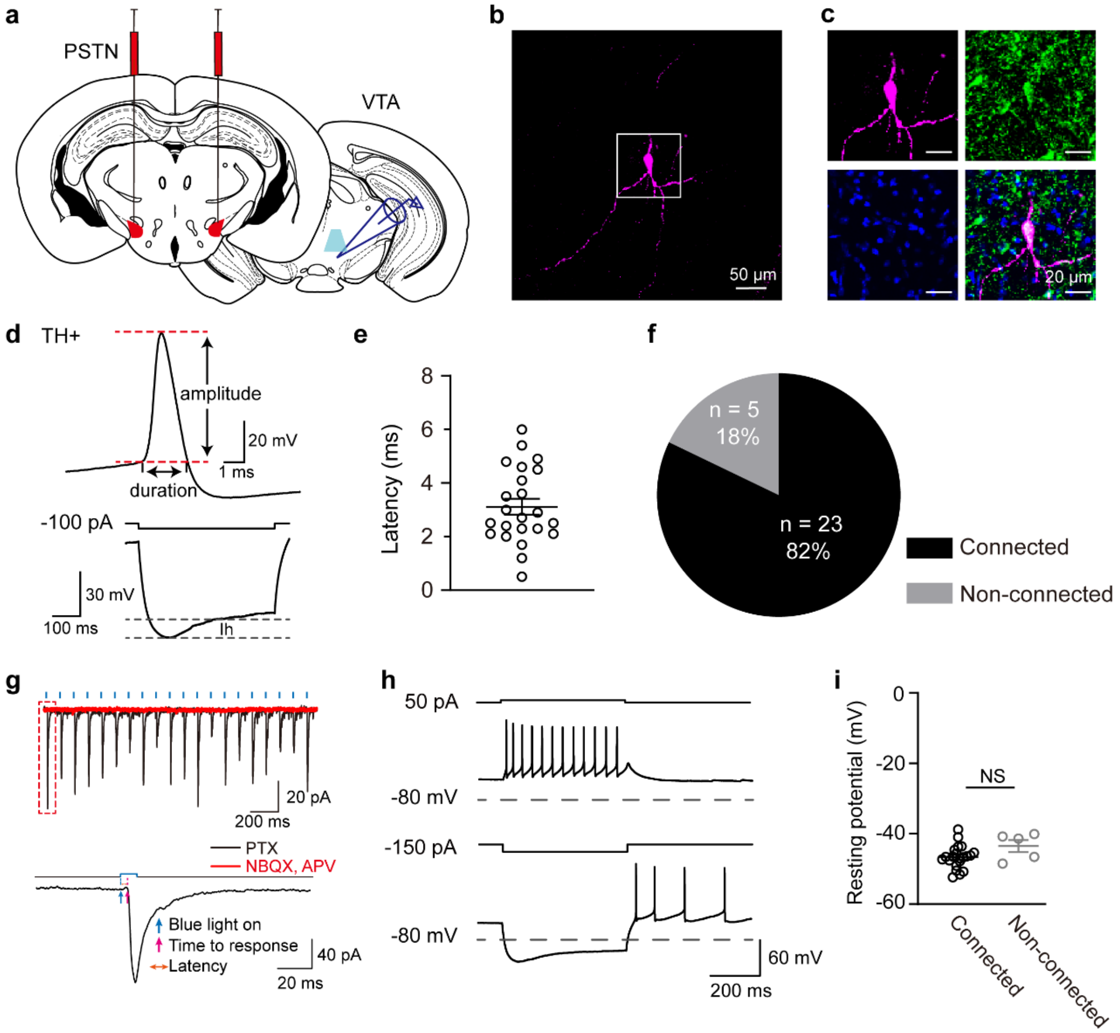

Supplementary Figure 8 PSTN CR neurons mainly innervated TH-positive VTA neurons.

a Schematic of the experiment. AAV-DIO-ChR2 was injected into the PSTN of CR-Cre mice, and the response was recorded in the VTA. **b** Representative picture of biocytin-labelled neurons (purple) recorded in the VTA. Scale bar: 50 μm. **c** Image of GFP (Th+, green), DAPI (blue), biocytin (purple), and merge showing a fluorescent view of the patched cell enlarged in the box of (**b**). Scale bar: 20 μm. **d** The electrophysiological characteristics of a TH-positive neuron. **e** Latency of photostimulation-evoked EPSCs averaged from 23 trials. **f** Number and proportion of recorded VTA dopaminergic neurons that responded and did not respond to the photostimulation of PSTN CR neuron terminals. **g** A typical example of a connected TH-positive-neuron responsive to light stimulation and light-evoked EPSCs were blocked by NBQX and APV, not PTX. **h** Typical traces of a VTA dopaminergic neuron in response to 50 and –150 pA current

injections. **i** Distribution of resting membrane potential of the connected and non-connected dopaminergic neurons in the VTA. Connected dopaminergic neurons: n=23 biologically independent cells; non-connected dopaminergic neurons: n=5 biologically independent cells. Unpaired two-sided *t-test* with no multiple comparisons; t_{26}=1.947, P=0.0624. Data represent mean ± SEM.

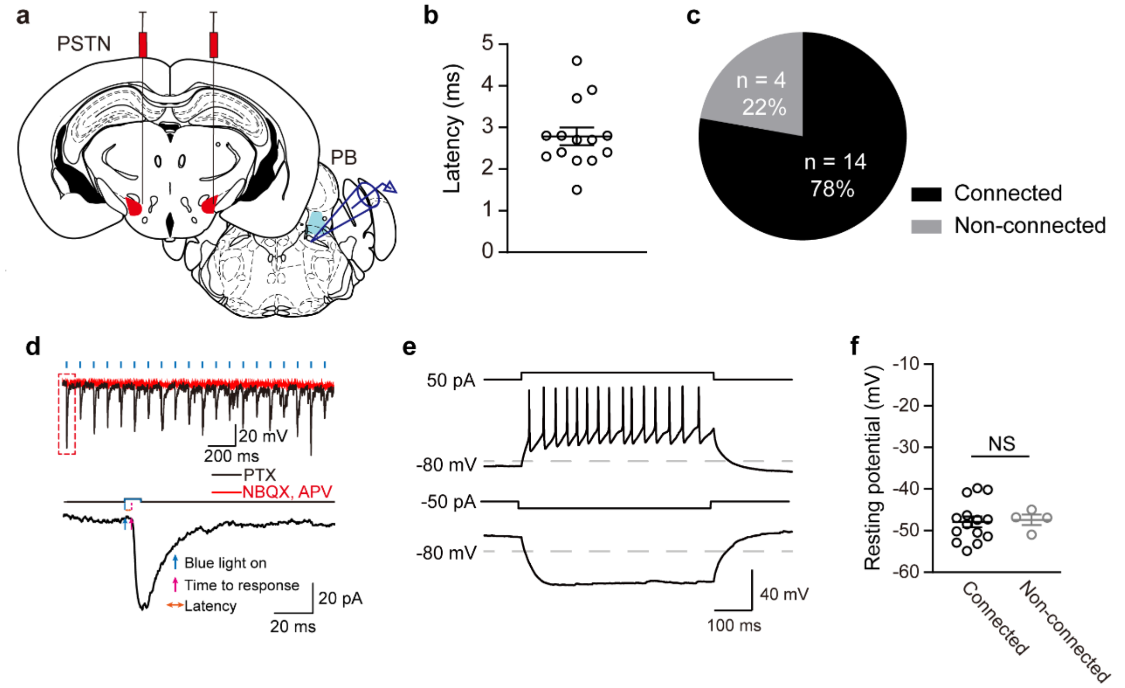

Supplementary Figure 9 PSTN CR neurons have monosynaptic functional connections with PB neurons.

a Schematic of experiment. AAV-DIO-ChR2 was injected into the PSTN of CR-Cre mice, and the response was recorded in the PB. **b** Latency of photostimulation-evoked EPSCs averaged from 14 trials. **c** Number and proportion of neurons recorded in the PB that responded and did not respond to the photostimulation of PSTN CR neurons terminals. **d** Photostimulation evoked EPSC in a neuron of PB. NBQX and APV, not PTX, abolished the EPSCs evoked by photostimulation. **e** Typical traces of a PB neuron in response to 50 and –50 pA current injections. **f** Distribution of the resting membrane potential of the connected and non-connected neurons in the PB. Connected neurons: n=14 biologically independent cells; non-connected neurons: n=4 biologically independent cells. Unpaired two-sided *t-test* with no multiple comparisons; t_{16}=0.1802, P=0.8593. Data represent mean ± SEM.

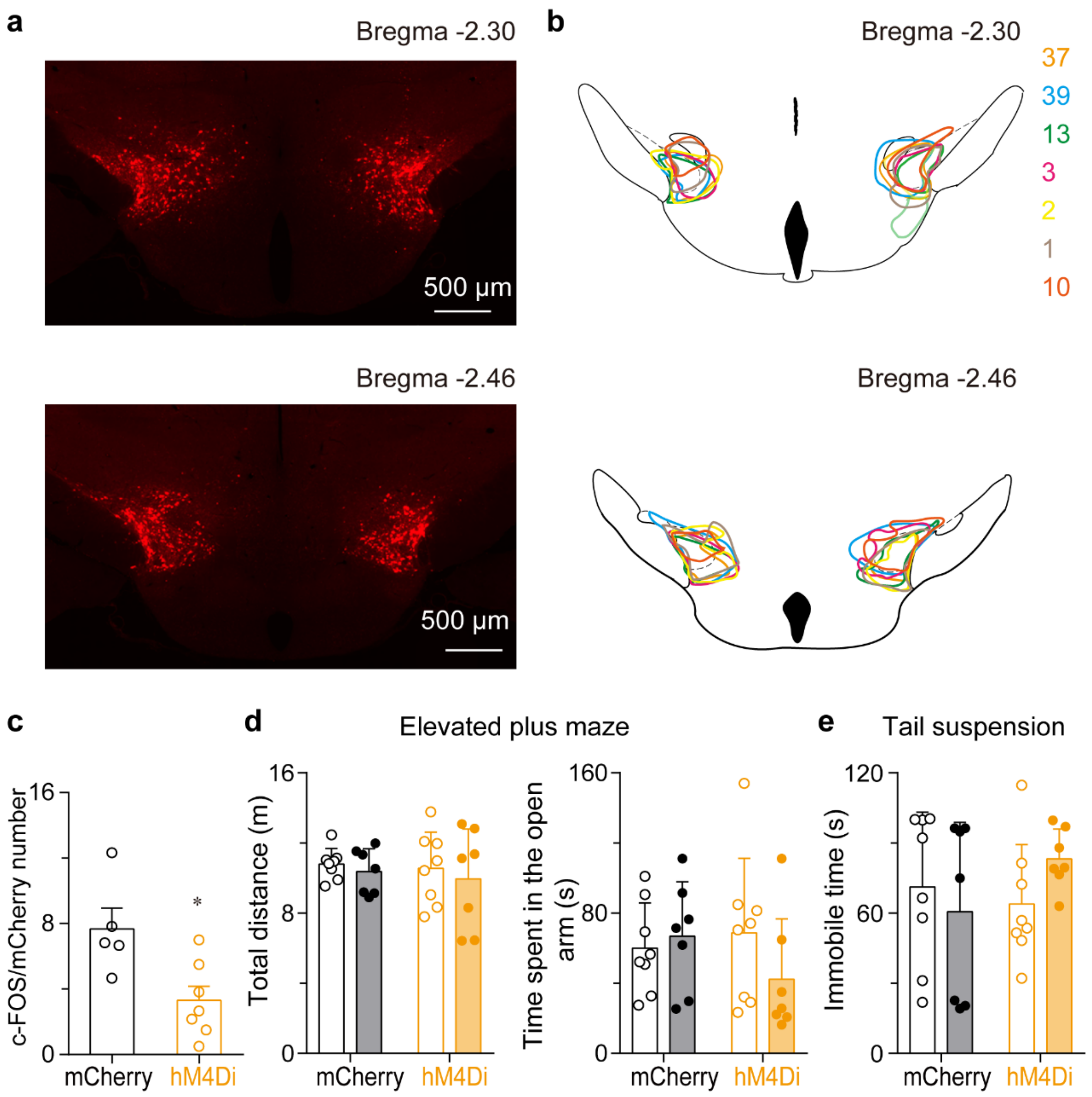

Supplementary Figure 10 Chemogenetic inhibition of PSTN CR neurons didn't affect behavior in the EPM and TST tests.

a Representative photomicrographs of the PSTN depicting mCherry (red) images of a PSTN-hM4Di mouse (Bregma=—2.30 in the upside and Bregma=—2.46 in the downside). **b** The drawings show superimposed AAV-hSyn-DIO-hM4Di-mCherry infection area in the PSTN of seven mice (Bregma=—2.30 in the upside and Bregma = –2.46 in the downside). **c** Quantification of c-Fos staining in mCherry or hM4Di-expressing neurons in the PSTN after CNO injection in mice (mCherry mice: n=5; hM4Di mice: n=7; unpaired two-sided *t-tests*; t_{10}=2.94, P=0.0148). **d, e** There were no change of behavior in the EPM and TST after inhibition of PSTN CR neurons (n = 7, unpaired two-sided *t-tests*). Data represent mean ± SEM, *P < 0.05.

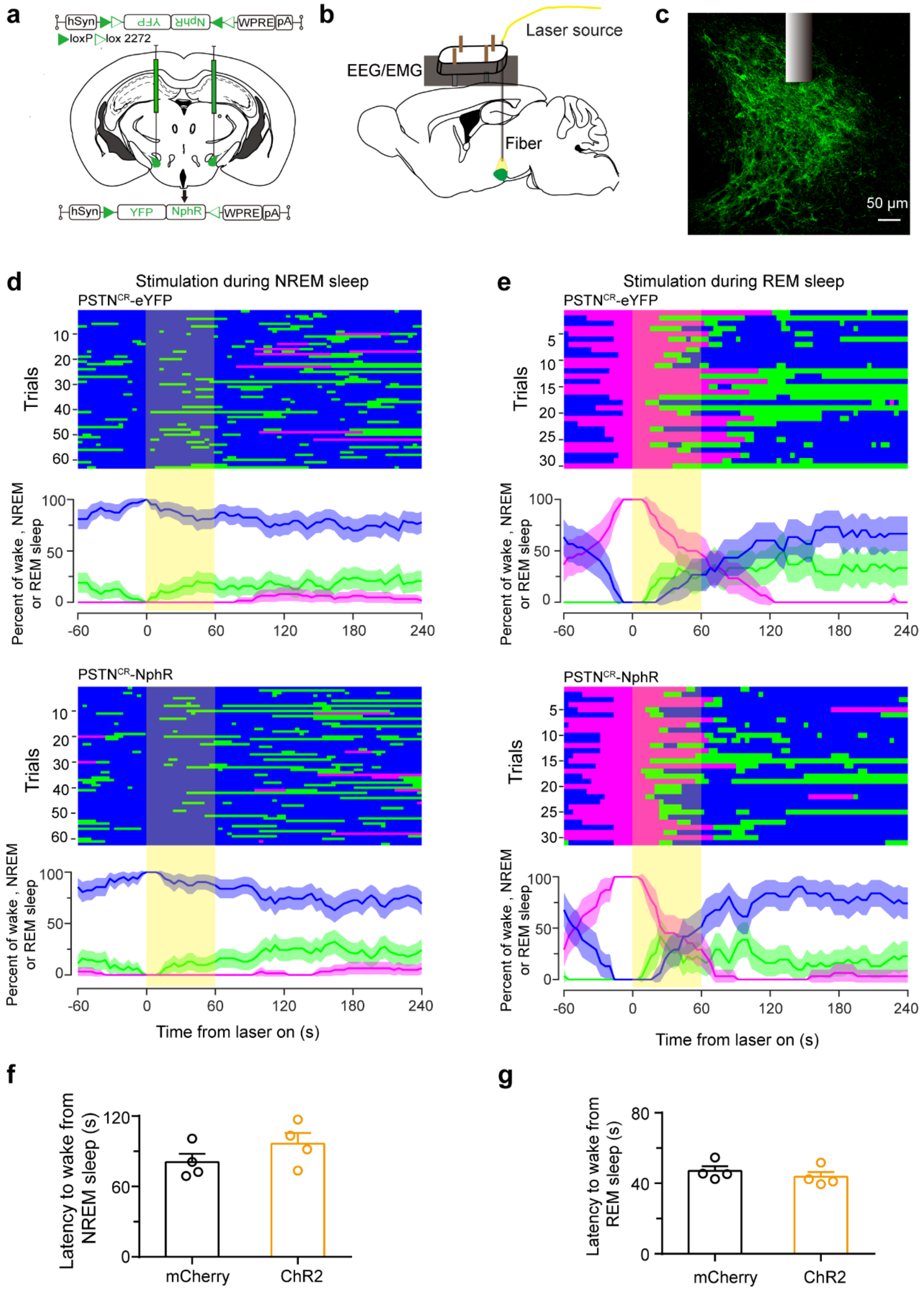

Supplementary Figure 11 Optogenetic inhibition of PSTN CR neurons during NREM sleep or REM sleep have little effect on transition.

a Diagram of unilateral viral infection area of AAV-hEF1a-DIO-NphR-eYFP. **b** Schematic of the *in vivo* optical stimulation. **c** Representative photomicrographs of the optical fiber location on the PSTN CR neurons expressing NphR-eYFP. Scale bar: 50 μm. **d** Illustration showing the 60 NREM sleep-to-wake transitions induced by photostimulation in eYFP (up) or NphR-eYFP (down) mice, n=4 per group. Quantification was based on an average of 10-20 stimulations per mouse. **e** Illustration showing the 30 REM sleep-to-wake transitions induced by photostimulation in eYFP (up) or NphR-eYFP (down) mice, n=4 per group. Quantification was based on an average of 5-10 stimulations per mouse. **f-g** Latencies of transitions from NREM (**f**) or REM (**g**) sleep to wakefulness after photostimulation (YFP group or NphR group: n=4, unpaired two-sided *t-test*). Data represent mean ± S.E.M.

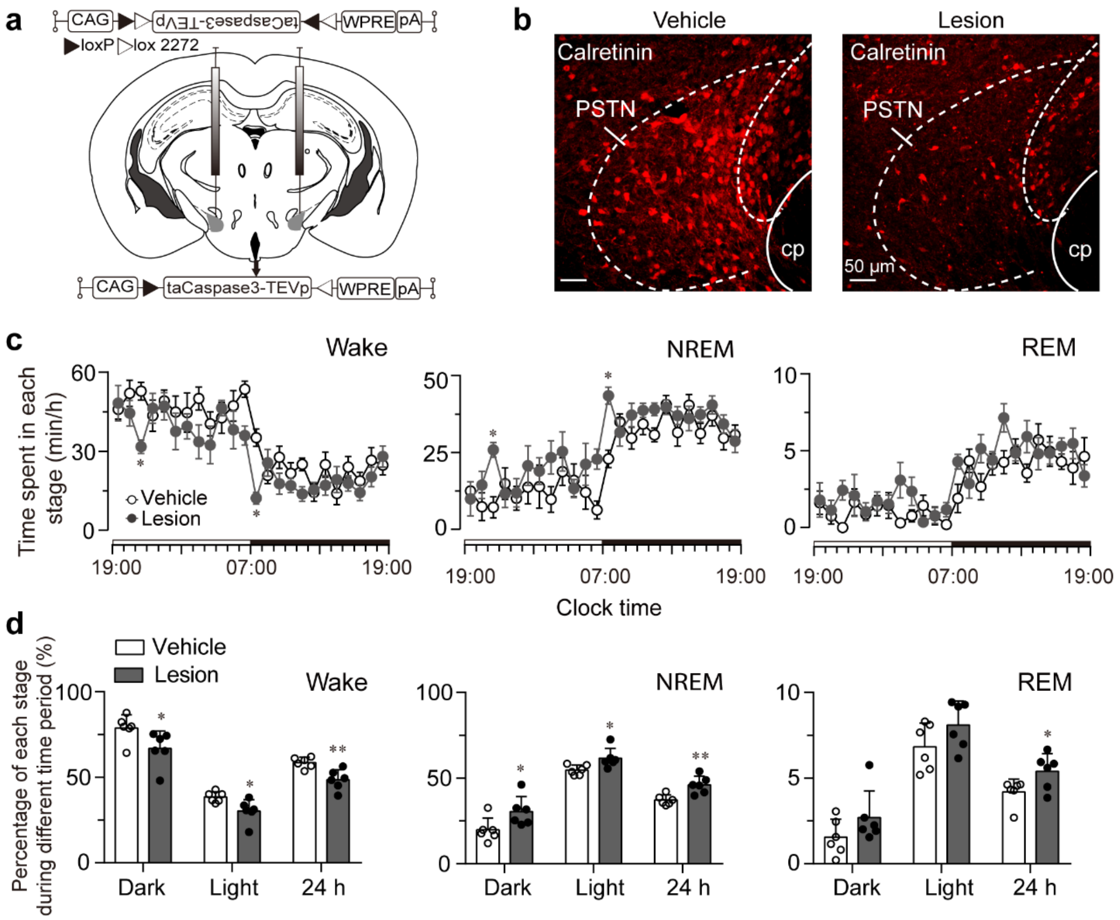

Supplementary Figure 12 Lesion of PSTN CR neurons decreased wakefulness in both dark and light phase.

a Diagram of unilateral viral infection area of AAV-CAG-DIO-taCaspase3. **b** Images showed that PSTN CR neurons were lesioned by caspase3. Scale bar: 50 µm. **c** Time spent in each stage across the 24 h sleep–wake cycle (Vehicle group or Lesion group, n= 6, Two-way ANOVA; Wake: $F_{1,10}$=13.63, P=0.0042; NREM: $F_{1,10}$=13.79, P=0.004; REM: $F_{1,10}$=5.436, P=0.0419). **d** The percentage of wakefulness, NREM sleep and REM sleep during different time period (Vehicle or Lesion group: n=6, unpaired two-sided *t-test*; Wake: t_{10}=2.27(Dark), 2.734(Light), 3.692(24 h), P=0.0466(Dark), 0.0211(Light), 0.0042(24 h); NREM: t_{10}=2.357 (Dark), 2.648(Light), 3.714(24 h), P=0.0402 (Dark), 0.0244 (Light), 0.004 (24 h); REM: t_{10}=1.508 (Dark), 1.592 (Light), 2.332 (24 h), P=0.1626 (Dark), 0.1424 (Light), 0.0419 (24 h)). Data represent mean ± SEM, $*P < 0.05$, $**P < 0.01$.

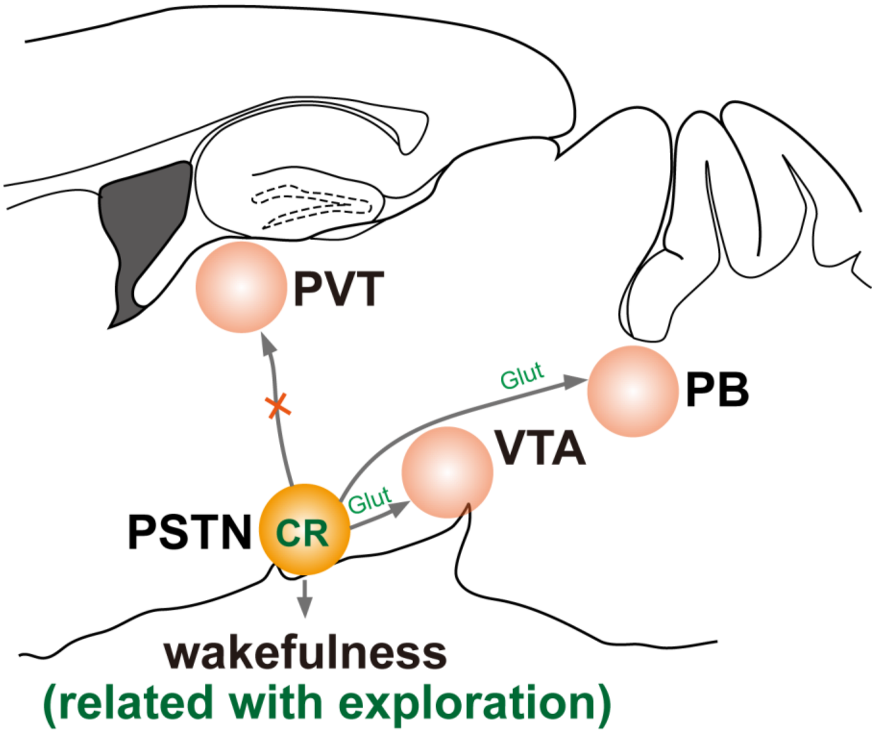

Supplementary Figure 13 Circuitry underlying the role of PSTN CR neurons in wakefulness regulation.

PSTN CR neurons play a pivotal role in regulating wakefulness associated with exploration. The PSTN CR neurons modulate wakefulness by releasing glutamate (Glut) transmitter to the VTA and PB, not the PVT. While exploration-related wakefulness induced by PSTN CR neurons is only through the PSTN^{CR}-VTA pathway.
