# Supplementary material for: Parasubthalamic calretinin neurons modulate wakefulness associated with exploration in male mice
Source: Nat Commun. 2023 Apr 24;14:2346. doi: 10.1038/s41467-023-37797-y (PMC10126000; doi:10.1038/s41467-023-37797-y)
Supplement: Supplementary file 2 — Reporting Summary [file 41467_2023_37797_MOESM2_ESM.pdf]

nature portfolio

Corresponding author(s): Zhili Huang

Last updated by author(s): Feb 18, 2023

Reporting Summary

Nature Portfolio wishes to improve the reproducibility of the work that we publish. This form provides structure for consistency and transparency in reporting. For further information on Nature Portfolio policies, see our Editorial Policies and the Editorial Policy Checklist.

Statistics

For all statistical analyses, confirm that the following items are present in the figure legend, table legend, main text, or Methods section.

n/a	Confirmed	
☐	☒	The exact sample size (*n*) for each experimental group/condition, given as a discrete number and unit of measurement
☐	☒	A statement on whether measurements were taken from distinct samples or whether the same sample was measured repeatedly
☐	☒	The statistical test(s) used AND whether they are one- or two-sided<br>*Only common tests should be described solely by name; describe more complex techniques in the Methods section.*
☐	☒	A description of all covariates tested
☐	☒	A description of any assumptions or corrections, such as tests of normality and adjustment for multiple comparisons
☐	☒	A full description of the statistical parameters including central tendency (e.g. means) or other basic estimates (e.g. regression coefficient) AND variation (e.g. standard deviation) or associated estimates of uncertainty (e.g. confidence intervals)
☐	☒	For null hypothesis testing, the test statistic (e.g. *F*, *t*, *r*) with confidence intervals, effect sizes, degrees of freedom and *P* value noted<br>*Give P values as exact values whenever suitable.*
☒	☐	For Bayesian analysis, information on the choice of priors and Markov chain Monte Carlo settings
☒	☐	For hierarchical and complex designs, identification of the appropriate level for tests and full reporting of outcomes
☒	☐	Estimates of effect sizes (e.g. Cohen's *d*, Pearson's *r*), indicating how they were calculated

Our web collection on statistics for biologists contains articles on many of the points above.

Software and code

Policy information about availability of computer code

Data collection	We collect the data of EEG/EMG signals using Vital Recorder software (At present, there is only one version. We checked the official website and there is no version number); the data of fiber signals by Spike2 software (CED, Cambridge, UK); the data of in vitro electrophysiology by pClamp10.3 software and the data of open-field test, elevated plus-maze test (EPM), light-dark box test, tail-suspension test by the Tracking Master V3.0 (TMV3) software, the data of immunohistochemical images by olympus microscope (VS-120, Tokyo, Japan), respectively.
Data analysis	We analyze the data of the EEG/EMG signal by SleepSign3-OBI software; the data of open-field test, elevated plus-maze test (EPM), light-dark box test, tail-suspension test by the Tracking Master V3.0 (TMV3) software; the data of in vitro electrophysiology by pClamp10.3 software; the data of mouse behavior in homecage by HomeCage Scan Version 3.00 software,and the data of immunohistochemical images by Photoshop cc(2018), respectively. We analyze the data of the fiber signal using a procedure written by MATLAB, the specific codes, deposited in the Figshare database (https://doi.org/10.6084/m9.figshare.22122179.v1). All data were statistical analyzed using GraphPad Prism 7 (GraphPad, USA).

For manuscripts utilizing custom algorithms or software that are central to the research but not yet described in published literature, software must be made available to editors and reviewers. We strongly encourage code deposition in a community repository (e.g. GitHub). See the Nature Portfolio guidelines for submitting code & software for further information.

Data

Policy information about availability of data

All manuscripts must include a data availability statement. This statement should provide the following information, where applicable:

- Accession codes, unique identifiers, or web links for publicly available datasets
- A description of any restrictions on data availability
- For clinical datasets or third party data, please ensure that the statement adheres to our policy

The source data generated in this study have been deposited in the Figshare database under accession code: https://doi.org/10.6084/m9.figshare.22121384.v1. The source data of the present study are provided.

Human research participants

Policy information about studies involving human research participants and Sex and Gender in Research.

Reporting on sex and gender	*Use the terms sex (biological attribute) and gender (shaped by social and cultural circumstances) carefully in order to avoid confusing both terms. Indicate if findings apply to only one sex or gender; describe whether sex and gender were considered in study design whether sex and/or gender was determined based on self-reporting or assigned and methods used. Provide in the source data disaggregated sex and gender data where this information has been collected, and consent has been obtained for sharing of individual-level data; provide overall numbers in this Reporting Summary. Please state if this information has not been collected. Report sex- and gender-based analyses where performed, justify reasons for lack of sex- and gender-based analysis.*
Population characteristics	*Describe the covariate-relevant population characteristics of the human research participants (e.g. age, genotypic information, past and current diagnosis and treatment categories). If you filled out the behavioural & social sciences study design questions and have nothing to add here, write "See above."*
Recruitment	*Describe how participants were recruited. Outline any potential self-selection bias or other biases that may be present and how these are likely to impact results.*
Ethics oversight	*Identify the organization(s) that approved the study protocol.*

Note that full information on the approval of the study protocol must also be provided in the manuscript.

Field-specific reporting

Please select the one below that is the best fit for your research. If you are not sure, read the appropriate sections before making your selection.

☒ Life sciences ☐ Behavioural & social sciences ☐ Ecological, evolutionary & environmental sciences

For a reference copy of the document with all sections, see nature.com/documents/nr-reporting-summary-flat.pdf

Life sciences study design

All studies must disclose on these points even when the disclosure is negative.

Sample size	We don't use statistical methods to predetermine the sample size. The sample size was determined empirically based on previous similar studies in the literature(PMID: 29679009). For chemogenetics, we determine the sample size as at least 7 mice. For optogenetics, we determine the sample size as at least 5 mice.
Data exclusions	For all experiments, mice with signs of unhealthy conditions after surgeries and inaccurate virus infection location were excluded for experiment.
Replication	For optical fiber calcium signal recording, experiments were replicated 5 times with independent mice. For chemogenetics, experiments were replicated 7-8 times with independent mice. For optogenetics, experiments were replicated at least 5 times with independent mice. For physiological recordings were replicated over 15 times in independent cells. For lesion study, experiments were replicated 6 times with independent mice. The reproducibility of microphages of optical fiber calcium recording, chemogenetics and optogenetics are consistent with the actual number of experimental animals. The repeatability of the micrograph for statistics is consistent with the number of animals. The repeatability of anterograde tracing experiments is at least three times.
Randomization	All experimental animals were randomly allocated into each experimental group.
Blinding	The investigators were blinded to group allocation during data collection and analysis.

Reporting for specific materials, systems and methods

We require information from authors about some types of materials, experimental systems and methods used in many studies. Here, indicate whether each material, system or method listed is relevant to your study. If you are not sure if a list item applies to your research, read the appropriate section before selecting a response.

Materials & experimental systems

n/a	Involved in the study
☐	☒ Antibodies
☒	☐ Eukaryotic cell lines
☒	☐ Palaeontology and archaeology
☐	☒ Animals and other organisms
☒	☐ Clinical data
☒	☐ Dual use research of concern

Methods

n/a	Involved in the study
☒	☐ ChIP-seq
☒	☐ Flow cytometry
☒	☐ MRI-based neuroimaging

Antibodies

Antibodies used

Rabbit polyclonal anti-c-Fos antibody, Millipore, Cat. #ABE457, lot number: 3168266.
Rabbit polyclonal anti-CR antibody, Swant, Cat. #7697, lot number: 1893-0114.
Rabbit polyclonal anti-mCherry antibody, Clontech, Cat. # 632496, lot number: 2103116.
Mouse monoclonal anti-NeuN, Millipore, Cat. #MAB377B, lot number: 3519281.
Donkey polyclonal anti-rabbit biotinylated IgG, Jackson ImmunoResearch, Cat. #711-065-152, lot number: 151508.
Donkey polyclonal anti-rabbit Alexa Fluor-conjugated 488 IgG antibody, Jackson ImmunoResearch, Cat. #711-545-152, lot number: 151331.
Donkey polyclonal anti-mouse Alexa Fluor-conjugated 594 IgG antibody, Jackson ImmunoResearch, Cat. #715-585-151, lot number: 155889.

Validation

The anti-c-Fos antibody is a rabbit polyclonal antibody for detection of c-Fos which has been validated in WB & IHC.
The anti-CR antibody is a rabbit polyclonal antiserum against calretinin which has been validated in WB & IHC. The antibody reacts specifically with calretinin in tissue originating from human, monkey, rat, mouse, guinea pig, chicken and fish.
The anti-NeuN antibody is a mouse monoclonal antibody for detection of NeuN which has been validated for use in IC, IH, IH(P), WB.
The anti-mCherry antibody is a polyclonal antibody for detection of mCherry which has been validated in WB, IHC and ELISA.
The donkey anti-rabbit biotinylated IgG reacts with whole molecule rabbit IgG and the light chains of other rabbit immunoglobulins.
The donkey anti-rabbit Alexa Fluor-conjugated 488 IgG antibody is a rabbit polyclonal antibody with well-characterized specificity for rabbit immunoglobulins and is excitated by the 488 nm laser line to detect its specified target.
The donkey anti-mouse Alexa Fluor-conjugated 594 IgG antibody is a mouse polyclonal antibody with well-characterized specificity for mouse immunoglobulins and is excitated by the 594 nm laser line to detect its specified target.

Animals and other research organisms

Policy information about studies involving animals; ARRIVE guidelines recommended for reporting animal research, and Sex and Gender in Research

Laboratory animals

We used adult male calretinin-IRES-Cre (C57BL/6) laboratory mice (8–12 weeks, 24–28 g) and Vglut2-IRES-Cre (C57BL/6) laboratory mice (8–12 weeks, 24–28 g) for experiments.

Wild animals

Our study did not involve wild animals.

Reporting on sex

All animals used in the experiment are male

Field-collected samples

Our study did not involve samples collected from the field.

Ethics oversight

All experimental protocols were approved by the Experimental Animal Ethics Committee of the School of Basic Medical Sciences, Fudan University (license identification number: 20200306-023).

Note that full information on the approval of the study protocol must also be provided in the manuscript.
